# Propolis: An update on its chemistry and pharmacological applications

**DOI:** 10.1186/s13020-022-00651-2

**Published:** 2022-08-26

**Authors:** Rajib Hossain, Cristina Quispe, Rasel Ahmed Khan, Abu Saim Mohammad Saikat, Pranta Ray, Damira Ongalbek, Balakyz Yeskaliyeva, Divya Jain, Antonella Smeriglio, Domenico Trombetta, Roghayeh Kiani, Farzad Kobarfard, Naheed Mojgani, Parvaneh Saffarian, Seyed Abdulmajid Ayatollahi, Chandan Sarkar, Mohammad Torequl Islam, Dılhun Keriman, Arserim Uçar, Miquel Martorell, Antoni Sureda, Gianfranco Pintus, Monica Butnariu, Javad Sharifi-Rad, William C. Cho

**Affiliations:** 1 Department of Pharmacy, Life Science Faculty, Bangabandhu Sheikh Mujibur Rahman Science and Technology University, Gopalganj, Dhaka 8100 Bangladesh; 2 Facultad de Ciencias de La Salud, Universidad Arturo Prat, Avda. Arturo Prat 2120, 1110939 Iquique, Chile; 3 Pharmacy Discipline, Life Science School, Khulna University, Khulna, 9280 Bangladesh; 4 Department of Biochemistry and Molecular Biology, Bangabandhu Sheikh Mujibur Rahman Science and Technology University, Gopalganj, 8100 Bangladesh; 5 Department of Biomedical Engineering, Huazhong University of Science and Technology, Wuhan, China; 6 Faculty of Chemistry and Chemical Technology, Al-Farabi Kazakh National University, 050040 Almaty, Kazakhstan; 7 Department of Bioscience and Biotechnology, Banasthali Vidyapith, Rajasthan 304022 India; 8 Department of Chemical, Biological, Pharmaceutical and Environmental Sciences (ChiBioFarAm), University of Messina, Viale Ferdinando Stagno d’Alcontres 31, 98166 Messina, Italy; 9 Department of Biology, Science and Research Branch, Islamic Azad University, Tehran, Iran; 10 Phytochemistry Research Center, Shahid Beheshti University of Medical Sciences, Tehran, Iran; 11 Department of Medicinal Chemistry, School of Pharmacy, Shahid Beheshti University of Medical Sciences, Tehran, Iran; 12 Department of Biotechnology, Razi Vaccine and Serum Research Institute, Agricultural Research, Education and Extension Organization (AREEO), Karaj, Iran; 13 Department of Pharmacognosy and Biotechnology, School of Pharmacy, Shahid Beheshti University of Medical Sciences, Tehran, Iran; 14 Food Processing Department, Vocational School of Technical Sciences, Bingöl University, Bingöl, Turkey; 15 Department of Nutrition and Dietetics, Faculty of Pharmacy, and Centre for Healthy Living, University of Concepción, Concepción, Chile; 16 Universidad de Concepción, Unidad de Desarrollo Tecnológico, UDT, 4070386 Concepción, Chile; 17 Research Group on Community Nutrition and Oxidative Stress, Laboratory of Physical Activity Sciences, and CIBEROBN - Physiopathology of Obesity and Nutrition, CB12/03/30038, University of Balearic Islands, Palma, Spain; 18 Department of Medical Laboratory Sciences, College of Health Sciences and Sharjah Institute for Medical Research, University of Sharjah, 22272 Sharjah, United Arab Emirates; 19 Department of Biomedical Sciences, University of Sassari, 07100 Sassari, Italy; 20 Chemistry & Biochemistry Discipline, University of Life Sciences King Mihai I from Timisoara, Calea Aradului 119, 300645 Timis, Romania; 21 Department of Clinical Oncology, Queen Elizabeth Hospital, Kowloon, Hong Kong

**Keywords:** Anticancer, Antioxidant, Anti-inflammatory, Bee glue, Bioactive compounds, Food preservative

## Abstract

Propolis, a resinous substance produced by honeybees from various plant sources, has been used for thousands of years in traditional medicine for several purposes all over the world. The precise composition of propolis varies according to plant source, seasons harvesting, geography, type of bee flora, climate changes, and honeybee species at the site of collection. This apiary product has broad clinical applications such as antioxidant, anti-inflammatory, antimicrobial, anticancer, analgesic, antidepressant, and anxiolytic as well asimmunomodulatory effects. It is also well known from traditional uses in treating purulent disorders, improving the wound healing, and alleviating many of the related discomforts. Even if its use was already widespread since ancient times, after the First and Second World War, it has grown even more as well as the studies to identify its chemical and pharmacological features, allowing to discriminate the qualities of propolis in terms of the chemical profile and relative biological activity based on the geographic place of origin. Recently, several in vitro and in vivo studies have been carried out and new insights into the pharmaceutical prospects of this bee product in the management of different disorders, have been highlighted. Specifically, the available literature confirms the efficacy of propolis and its bioactive compounds in the reduction of cancer progression, inhibition of bacterial and viral infections as well as mitigation of parasitic-related symptoms, paving the way to the use of propolis as an alternative approach to improve the human health. However, a more conscious use of propolis in terms of standardized extracts as well as new clinical studies are needed to substantiate these health claims.

## Introduction

The name "propolis" derives from two terms of Greek origin, “pro” and “polis”, which literally mean "in favor of the city" [1]. In fact, propolis, which is a sticky, gummy, and balsamic material collected from plants, is used by bees (*Apis mellifera* L.) to coat the hive and protect it from diseases caused by fungi, yeast, and bacteria as well as from predators [2]. In particular, propolis derives from a resin that is found mainly in the buds and bark of poplars, birches and conifers in general. The foraging bees collect these resins obtaining a mixture of resinous substances, pollen, waxes, and enzymes [3, 4]. Propolis is commonly employed by bees as building material and sealer [5] by maintaining homeostasis, reducing vibrations, keeping airflow, defend the colony against squatters, and prevent putrefaction [6]. It consists of granules of various sizes and shades of color (yellow, red, and dark brown) depending on its botanical source, while its smell is strongly aromatic [7]. Its consistency is hard and crumbly in nature, but as soon as it is handled and slightly heated, it becomes viscous and sticky, melting at temperatures around 70 °C [8].

Propolis has been the topic of several research conducted around the world in recent decades, and its chemical composition and biological properties have been widely explored [9–15]. The most common uses of propolis are as immunostimulant, as an aid to prevent colds thanks to its antibacterial and antiviral action, as a natural remedy in case of skin problems due to its soothing and healing effect, also in the oral cavity to treat small ulcers and canker sores, to relieve redness and itching of the urinary tract and finally to restore the balance of the gastric mucosa [16–18].

Recently, some mechanisms of action have been suggested paving the way to new clinical applications. The aim of this study is to provide a comprehensive review of these recent findings, discussing the current state of propolis research as well as the future prospectives.

## History of propolis

In the long and rich history of human beekeeping, apiary products have been widely used due to their recognized beneficial properties. In particular, the traditional use of propolis is known since 300 B.C. The Egyptians worshiped the bee and used propolis in the mummification process [19]. It was also well known to the priests who, at that time, monopolized medicine and chemistry [1]. Bees played an essential role in Greek and Roman religious traditions assuming many symbolic meanings and being featuring in many stories of the Greek and Roman gods. Among them, one tells of Zeus (Jupiter for Romans), who, to escape his father, the god Cronus, was hidden in a secret cave by his mother and fed thanks to the honey of the sacred bees. Probably due to this story, one of Zeus' names was "*Melissaios*", or "bee-man". According to this legend, it was Zeus himself, who gave the bees their bright gold color and to made them strong enough to withstand the cold and winds.

In traditional Georgian medicine, some ailments were treated using propolis ointments. There was also the custom of putting a propolis cake on the navel of the newborn. During the Anglo-Boer War [2] and World War II, doctors used propolis to heal wounds efficiently. Finally, in the USRR, the orthodox medicine recognized the therapeutic use of propolis (30% alcohol solution) already in 1969 [20]. Propolis was a key component in the Greek fragrance polyanthus, which included propolis, olibanum, styrax, and fragrant plants [21]. It has been extolled by more than 15 Greek and Roman writers (beside honey and wax) such as Aristotele in its “*Historia Animalium*” and Hippocrates, who is known to have employed propolis to treat wounds and ulcers [21]. Moreover, Dioscorides, in his main book “*De materia Medica*”, outlined the medicinal applications of propolis, by citing also honey, wax, and different honey wines as medicines [22]. The bee and propolis were likewise highly esteemed by the Romans. Just think of Pliny the Elder, who discussed it, enhancing its multiple therapeutic applications as purifying agent, tumors dispersant, calming agent for tendon pains and for its healing properties [23].

## Traditional uses

Propolis has long been used as a bactericidal, antiviral, and antifungal drug in folk medicine to treat inflammations in several body areas worldwide [24]. It was used for skin regeneration, wound healing, and as local anesthetic and, in this sense, it was found in nearly all home first-aid kits [25]. Propolis has also been advised in folk medicine for the treatment of purulent disorders, as it has been shown to improve wound healing and relieve many types of discomfort. Craftsmen utilized propolis also for no-health purposes such as windows sealer, impregnant for valuable timber objects, varnish and repairing instrument [26]. The alternative and complementary medicine used different propolis-based preparations such as sprays, ointments, and powders (mainly consisting of tinctures and ethanolic extracts) for the treatment of colds, flu, bronquial asthma, and other human ailments [27] such as gastric disorders [28]. Moreover, propolis is still used as an active substance in some dietary supplements, cosmetics, and even medicinal sweets. Unlike honey and bee pollen, it has no nutritional value, but exerts a very strong and multidirectional biotic effect [29]. Propolis has recently become popular in food and beverages as a way to boost health and prevent illness [30, 31]. It is still used to treat wounds and burns, as well as sore throats, dental caries, and stomach ulcers [32]. For years, propolis ethanolic extract has been known to have anti-inflammatory properties and used as an immunomodulatory agent [24, 33]. It can be used for various purposes in endodontics and would have a promising role in dentistry [28]. Researchers used nanoparticles to test the usage of propolis for various purposes. The use of nanoparticle-based delivery methods has the potential to make hydrophobic compounds like propolis dispersible in aqueous media, avoiding the problems associated with poor solubility [34].

## Chemical composition and physical properties

**Fig. 1:**
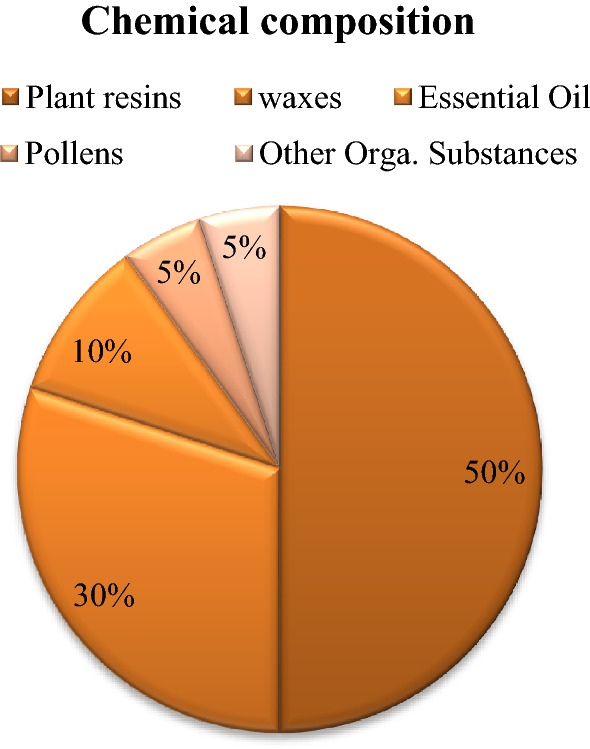
Chemical composition (%) of raw propolis

It is well-known that a medicinal plant demonstrates a pharmacological effect thanks to its chemical constituents. Raw propolis contains not only plant resins, but also waxes, essential oil, pollens, and other organic sunstance in different percentage as depicted in Fig. 1. Thanks to this multitude of components, it consists of a rather complex chemical profile as reported in Table 1. Many studies reported that propolis contains, in particular, phenolic acids, flavonoids, ketones, aldehydes, chalcones, dihydrochalcones, terpenoids, amino acids, aliphatic acids, aromatic esters and acids, carbohydrates, vitamins, metals, and also beeswax [35–40].

**Table 1:** Chemical composition of raw propolis

Class	Compounds
Alcohols	Benzene methanol, cinnamyl alcohol, glycerol; a-glycerophosphate; hydroquinone; isobutenol, phenethyl alcohol; prenyl alcohol [176, 177, 353]
Aldehydes	Benzaldehyde; caproiealdehyde; *p-*hydroxybenzaldehyde; isovanillin; protocatechuic aldehyde; vanillin [176, 353–356]
Aliphatic acids and aliphatic esters	Acetic acid; angelic acid; butyric acid; crotonic acid; fumaric acid; isobutyric acid; methylbutyric acid; isobutyl acetate; isopentyl acetate; isopentenyl acetate [176]
Amino acids	Alanine; β-alanine; α-aminobutyric acid; 6-aminobutyric acid; arginine; asparagine; aspartic acid; cystine; cysteine; glutamic acid; glycine; histidine; hydroxyproline; isoleucine; leucine, lysine; methionine; ornithine; phenylalanine; proline; pyroglutamic acid, sarcosine; serine; threonine, tryptophan; tyrosine; valine [49, 50, 176, 357]
Aromatic acid	*p*-Anisic acid; benzoic acid; caffeic acid; cinnamic acid; coumaric (-*o*,-*m*,-*p*) acid; 3,4-dimethoxycinnamic acid; ferulic acid; gallic acid; gentisic acid; hydroxycinnamic acid; *p-* hydroxybenzoic acid; isoferulic acid; 4-methoxycinnamic acid; protocatechuic acid; salicylic acid; vanillic acid; veratric acid [176, 177, 353–356, 358, 359]
Aromatic esters	Benzyl acetate, benzyl benzoate, benzyl caffeate, benzyl coumarate; benzyl-3,4-dimethoxycinnamate; benzyl ferulate; benzyl isoferulate; benzyl salicylate; butenyl caffeate; butyl caffeate; cinnamyl benzoate; cinnamyl caffeate; butyl caffeate; cinnamyl coumarate; cinnamyl isoferulate; ethyl benzoate; ethyl caffeate; methyl benzoate; 2-methyl-2-butenyl caffeate; 3-methyl-2-butenyl caffeate; 3-methyl-3-butenyl caffeate; 3-methyl-3-butenyl coumarate; 3-methyl-2 -butenyl ferulate; 3-methyl -3-butenyl ferulate; 2-methyl-2-butenyl isoferulate, 3-methyl-3-butenyl isoferulate; methyl salicylate; phenyl ethyl caffeate: phenyl ethylcoumarate; phenyl–ethyl isoferulate; pentyl caffeate; pentenyl caffeate, pentenyl ferulate; prenyl caffeate; prenyl coumarate; prenyl ferulate; prenyl isoferulate [176, 177, 353, 358, 359]
Chalcones and dihydrochalcones	Alpinetin chalcone; naringenin chalcone; pinobanksin chalcone, pinobanksin-3-acetate chalcone; pinocembrin chalcone; pinostrobin chalcone; sakuranetin chalcone; 2',4’,6' -trihydroxy-4'-methoxychalcone; 2',6'-dihydroxy-4'-methildihydrochalcone; 2’,4',6’- trihydroxydihydrochalcone [176, 177, 353, 359]; 3,4,2',3'-tetrahydroxychalcone [360], isoliquiritigenin, 4,4'-dihydroxy-2'-methoxychalcone; 4,2',4',alpha-tetrahydroxydihydrochalcone, 2',4'-dihydroxychalcone [361]
Flavanones	Naringenin; pinobanksin; pinobanksin-3-acetate; pinobanksin-3-butyrate; pinobanksin-3-hexanoate, pinobanksin-3-methyl ether; pinobanksin-3-pentanoate; pinobanksin-3-pentenoate; pinobanksin-3-propanoate; pinocembrin; pinostrobin; sakuranetin; 3,7-dihydroxy-5-methoxytlavanone; 2,5-dihydroxy-7-methoxyflavanone [49, 176, 177, 355, 359, 362–364]; 5,7-dihydroxy-6-methoxy-2,3-dihydroflavonol-3-acetate [365], 5-methoxy-3-hidroxyflavanone [366], (2*R*, 3*R*)-3,6,7-trihydroxyflavanone [367], alnustinol, (2*R*,3*R*)-3,7-dihydroxy-6-methoxyflavanone, garbanzol, (2*R*,3*R*)-3,7-dihydroxyflavanone, (2*S*)-dihydrooroxylin A, (2*S*)-dihydrobaicalein, (2*S*)-nNaringenin, (2*S*)-7-hydroxy-6-methoxyflavanone, (2*S*)-liquiritigenin, (2*S*)-7-hydroxyflavanone [361], sophoraflavanone A, solophenol A, bonannione A [368], sigmoidin B, propolin E, propolin B, propolin A [369], 5,7,3',4'-tetrahydroxy-2'-C-geranyl-6-prenlyflavanone, 5,7,3',4'-tetrahydroxy-2'-*C*-geranylflavanone, 5,7,3',4'-tetrahydroxy-6-*C*-geranylflavanone, 5,7,3',4'-tetrahydroxy-5'-*C*-geranylflavanone [48]; 3',4',6-trihydroxy-7-methoxy flavanone [367], (2*R*,3*R*)-6[1-(4'-hydroxy-3'-methoxyphenyl) prop-2en-1-yl]-pinobanksin-3-acetate, (2*R*,3*R*)-6[1-(4'-hydroxy-3'-methoxyphenyl) prop-2en-1-yl] pinobanksin [370], (2*R*,3*R*)-3,5-dihydroxy-7-methoxyflavanone 3-(2-methyl)-butyrate [97]; 7-*O*-prenylpinocembrin, 7-*O*-prenylstrobopinin [371]; pinobanksin-5-methyl-ether-3-*O*-pentanoate, hesperitin-5,7-dimethyl ether [366], (2*S*)-5,7-dihydroxy-4'-methoxy-8-prenylflavanone [368], 3-*O*-[(*S*)-2-methylbutyroyl] pinobanksin [372]
Flavones and flavonols	Acacetin; apigenin; apigenin-7-methyl ether; chrysin, fisetin; galangin; galangin-3-methyl ether; izalpinin; isorhamnetin; kaempferide; kaempferol; kaempterol-3-methyl ether; kaempferol-7-methyl ether; kaempferol-7,4’-dimethyl ether; pectolinarigenin; quercetin; quercetin-3,7-dimethyl ether; ramnetin; ramnocitrin, tectocrisin [49, 176, 177, 353–356, 364]; luteolin [373]; 6-cinnamylchrysin [372]; 3',5-dihydroxy-4',7-dimenthoxy flavones [374]; hexamethoxy flavones [375]; (7''*R*)-8-[1-(4'-hydroxy-3'-methoxyphenyl) prop-2-en-1-yl] chrysin [97]; (7"*R*)-8-[1-(4'-hydroxy-3'-methoxyphenyl)prop-2-en-1-yl]-galangin [97]; macarangin [376]; 2'-geranylquercetin, 8-(8"-hydroxy-3",8"-dimethyl-oct-2"-enyl)-quercetin, 2'-(8"-hydroxy-3",8"-dimethyl-oct-2"-enyl)-quercetin [368]
Isoflavones	4',7-dimethoxy-2'-isoflavonol, medicarpin, homopterocarpin, 7,4'-dihydroxyisoflavone [377], calycosin [361], 5,7-dihydroxy-4'-methoxyisoflavonoid, 7-hydroxy-4'-methoxyisoflavonoid [378], 7,3'-dihydroxy-6,5'-methoxyisoflavonoid, 6,7,3'-trihydroxy-4'-methoxyisoflavonoid, 7,3',4'-trihydroxy-5'-methoxyisoflavonoid, odoratin [367]; biochanin [379], (3*R*)-4'-Methoxy-2',3,7-trihydroxyisoflavanone, (3*S*)-ferreirin, (3*S*)-violanone, (3*S*)-vestitone, 2'-hydroxybiochanin A, pratensein, biochanin A, xenognosin B, formononetin, daidzein [361]
Flavans and Isoflavans	8-[(*E*)-4-phenylprop-2-en-1-one]-(2*R*,3*S*)-2-(3,5-dihydroxyphenyl)-3,4-dihydro-2H-2-be-nzopyran-5-methoxyl-3,7-diol, 8-[(*E*)-4-phenylprop-2-en-1-one]-(2*S*,3*R*)-2-(3,5-dihydroxyphenyl)-3,4-dihydro-2H-2-benzopyran-5-methoxyl-3,7-diol, 8-[(*E*)-4-phenylprop-2-en-1-one]-(2*R*,3*S*)-2-(3-methoxyl-4-hydroxyphenyl)-3,4-dihydro-2H-2-benzopyran-5-methoxyl-3,7-diol [380]; 3-hydroxy-5,6-dimethoxyflavan [381]; (3*S*)-vestitol, (3*S*)-isovestitol, (3*S*)-7-*O*-methylvestitol, (3*S*)-mucronulatol [361]; 7,4'-dihydroxy-2'-methoxyisoflavone [378]; neovestitol [379]
Open-chain neoflavonoids and other flavonoids	(S)-4-methoxydalbergione, (*S*)-3',4'-dihydroxy-4-methoxydalbergione, (*S*)-3'-hydroxy-4-methoxydalbergione [367], neoflavonoid 1–10 [382], (*Z*)-1-(2'-methoxy-4',5'dihydroxyphenyl)-2-(3-phenyl)propene, 1-(3',4'-dihydroxy-2'-methoxyphenyl)-3-(phenyl)propane [381], 2-(2',4'-dihydroxyphenyl)-3-methyl-6-methoxybenzofuran, 2,6-dihydroxy-2-[(4-hydroxyphenyl)methyl]-3-benzofuranone [361]
Hydrocarbon esters ethers, hydroxyl and keto waxes	Heneicosane; hentriacontane; heptacosane; hexacosane; nonacosane; pentacosane; tricosane; tripentacontane; tritriacontane; dotriacontyl hexadecanoate; dotriacontyl-[(Z)-octadec-9-enoate]; hexacosyl hexadecanoate; hexacosyl-[(Z)-octadec-9-enoate]; octacosylhexa decanoate; octacosyl-[(Z)-octadec-9-enoate] tetracosyl-hexadecanoate; tetracosyl-[(Z)-octadec-9-enoate]; tetratriacontyl-hexadecanoate tetratriacontyl-[(Z)-octadec-9-enoate]; triacontyl-hexadecanoate; triacontyl–[(Z)-octadec-9-enoate] [177, 353, 383, 384]
Pterocarpins (a type of neoflavonoid)	(6a*R*,11a*R*)-4-methoxymedicarpin, 6a-ethoxymedicarpin, 3,10-dihydroxy-9-methoxypterocarpan [361], 3,4-dihydroxy-9-methoxypterocarpan, 3-hydroxy-8,9-dimethoxypterocarpan, 3,8-dihydroxy-9-methoxypterocarpan, 4'-methoxy-5'hydroxyvesticarpan, homopterocarpin, 4-hydroxymedicarpin, medicarpin [378]
Fatty acids	Arachid acid; behenic acid; cerotic acid; lauric acid; linoleic acid; lignoceric acid; montanic acid; myristic acid; oleic acid; palmitic acids; stearic acid [177, 353, 359, 383]
Ketones	Acetophenone; *p-*acetophenolacetophenone; dihydroxy-acetophenone; methylacetophenone; hept-5-en-2-one; 6-methylketone [177, 353, 359]
Terpenoids and other compounds	α-Acetoxibetulenol; β-bisabolol; 1,8-cineole; α-copaene; cymene; limonene; pterostilbene; styrene; xanthorreol; xylitol; naphthalene; 4-hexanoIactone; sesquiterpene alcohol; sesquiterpene diol [49, 177, 353, 354, 363]; linalool [385], trans-β-terpineol [386], camphor [387]; junipene [386], γ-elemene, α-ylangene, valencene [385], 8-βH-cedran-8-ol, 4-βH,5α-eremophil-1(10)-ene, α-bisabolol, α-eudesmol, α-cadinol [388], patchoulene, manoyl oxide, ferruginol, ferruginolone, 2-hydroxyferruginol, 6/7-hydroxyferruginol, sempervirol, abietic acid, 18-succinyloxyabietadiene, 18-succinyloxyhydroxyabietatriene, 18-hydroxyabieta-8,11,13-triene, imbricataloic acid, diterpenic acid, neoabietic acid, labda-8(17),12,13-triene, hydroxydehydroabietic acid, dihydroxyabieta-8,11,13-triene, 13(14)-dehydrojunicedric acid, dehydroabietic acid, 18-hydroxyabieta-8,11,13-triene [389], junicedric acid, 14,15-dinor-13-oxo-8(17)-labden-19-oic acid, tran-communal, palmitoyl isocupressic acid, oleoyl isocupressic acid, 13-hydroxy-8(17),14-labdadien-19-oic acid, 15-oxolabda-8(17),13(E)-dien-19-oic acid, pimaric acid, totarolone [390]; lupeol alkanoates, lupeol, 24-methylene-9,19-ciclolanostan-3β-ol [391], lupeol acetate, lanosterol, germanicol acetate, germanicol, β-amyrin acetate, β-amyrone, α-amyrin acetate, α-amyrone [392], lanosterol acetate [375], (22Z,24E)-3-Oxocycloart-22,24-dien-26-oic acid, (24E)-3-oxo-27,28-dihydroxycycloart-24-en-26-oic acid [393], 3,4-seco-cycloart-12-hydroxy-4(28),24-dien-3-oicacid, cycloart-3,7-dihydroxy-24-en-28-oic acid [390], 3-oxo-triterpenic acid methyl ester [394]; 2*H*-cyclopentacyclooctene,4,5,6,7,8,9-hexahydro-1,2,2; 3-tetramethyl; germanicol; dimethyl-1,3,5,6-tetramethyl-[1,3-(13C2)] bicycle; dodeca-1,3,5,6,8,10-hexaene-9,10-dicarboxylate; spiro[benzo[α]cyclopenta [3, 4]cyclobuta[1,2-c]cycloheptene-8(5*H*),2'-[1,3]dioxane]; 6,7,7β,10α-tetrahydro-1; 14-methyl-cholest-7-en-3-ol-15-one; (3α,4α)- 4-methyl-stigmast-22-en-3-ol [28]
Lignans	Tetrahydrojusticidin B, 6-methoxydiphyllin, phyllamricin C [376]
Phenylpropanoids	*cis*-3-Methoxy-4-hydroxycinnamic acid, *trans*-3-methoxy-4-hydroxycinnamic acid [395]; 3-prenyl cinnamic acid allyl ester, *p*-methoxycinnamic acid, dihydrocinnamic acid, 3-methyl-2-butenyl isoferulate, 3-methyl-3-butenyl caffeate, hexadecyl caffeate [396]; 3-prenyl-4-hydroxycinnamic acid, 3,5-diprenyl-4-hydroxycinnamic acid [397]; methyl(*E*)-4-(4'-hydroxy-3'-methylbut-(*E*)-2'-enyloxy) cinnamate [398]; tetradecenyl caffeate (isomer), tetradecenyl caffeate [375]; 2-methyl-2-butenyl ferulate [48]
Chlorogenic acids	4-Feruoyl quinic acid [399]; 5-ferruoyl quinic acid [373]; 3,4,5-tri-*O*-caffeoylquinic acid [400]
Stilbenes	5'-Farnesyl-3'-hydroxyresveratrol [368]; schweinfurthin A, B [376]; 5,4'-dihydroxy-3'-methoxy-3-prenyloxy-*E*-stilbene, 3,5,3',4'-tetrahydroxy-2-prenyl-*E*-stilbene, 3,5,4'-trihydroxy-3'-methoxy-2-prenyl-*E*-stilbene, 5,3',4'-trihydroxy-3-methoxy-2-prenyl-*E*-stilbene, 5,4'-dihydroxy-3,3'-dimethoxy-2-prenyl-*E*-stilbene, 5,4'-dihydroxy-3-prenyloxy-*E*-stilbene, 3',4'-dihydroxy-*E*-stilbene, 3',4'-dihydroxy-3,5-dimethoxy-*E*-stilbene, diprenylated dihydrostilbene, 3,5-dihydroxy-2-prenyl-*E*-stilbene, 4-prenyldihydroresveratrol, 3-prenylresveratrol [398]; ( +)-pinoresinol dimethyl ether, ( +)-pinoresinol, ( +)-syringaresinol [361]
Other phenolics	8-(Methyl-butanechromane)-6-propenoic acid, 3-hydroxy-2,2-dimethyl-8-prenylchromane-6-propenoic acid, 2,2-dimethyl-8-prenylchromene-6-propenoic acid, 2,2-dimethylchromene-6-propenoic acid, 2,2-dimethyl-6-carboxyethnyl-2*H*-1-benzopyran, 2,2-dimethyl-6-carboxyethenyl-8-prenyl-2*H*-1-benzopyran [401]; nemorosone, 7-epi-clusianone, xanthochymol, gambogenone, hyperibone A [402]; 5-pentadecylresorcinol, 5-(8'*Z*,11'*Z*-heptadecadienyl)-resorcinol, 5-(11'*Z*-heptadecenyl)-resorcinol, 5-heptadecylresorcinol [95]; 1,3-bis(trimethylsilylloxy)-5,5-proylbenzene, 3,4-dimethylthioquinoline, 4-oxo-2-thioxo-3-thiazolidinepropionic acid, D-glucofuranuronic acid, dofuranuronic acid, 3-quinolinecarboxamine [403], baccharin [20], suberosin, tschimgin, tschimganin, bornyl *p*-hydroxybenzoate, bornyl vanillate, ferutinin, tefernin, ferutinol *p*-hydroxybenzoate, ferutinol vanillate [387]; 2-acetoxy-6-*p*-methoxybenzoyl jaeschkeanadiol, 2-acetoxy-6-*p*-hydroxybenzoyl jaeschkeanadiol [404]
Steroids	Calinasterol acetate; β-dihydrofucosterol acetate; ucosterol acetate; stigmasterol acetate [178]
Sugars	Fructofuranose-1; fructofuranose-2; α-D-glucopyranose; β-D-glucopyranose [177, 353, 358]; galactitol, gluconic acid, galacturonic acid and 2-*O*-glycerylgalactose [394]

The color and melting point of propolis varies according to area and the plant source [4]. Propolis melts on 60 °C to 70 °C while some of its kinds melt on 100 °C [4, 26]. Ethanol is the best suitable solvent to obtain commercially extracts of propolis but is also used methanol, chloroform, ether and acetone [4, 26, 41].

## Compounds isolated from propolis

According to literature up to now about 300 different chemicals components have been determined in propolis of different sources [4, 9, 42–44]. Moreover, the major chemical constituents are varied in amount and type and depend on the extraction process associated with the extraction solvents. Recently, Bankova et al. [45] reviewed classical and modern methods of extraction of propolis such as maceration, Soxhlet, ultrasound-assisted and microwave-assisted extraction, supercritical CO_2_ extraction, high-pressure methods, and the application of different solvents. The authors concluded that ultrasound-assisted extraction is the best optimal method, considering into account extraction time and extraction yield, and concerning the solvents, mixtures of water and ethanol are most effective.

Propolis has a very rich and complex chemical composition, which varies according to several parameters such as plant source, seasons harvesting, geography, type of bee flora, climate changes, and honey bee species at the collection site [15, 44, 46, 47]. Indeed, more than 300 different compounds have been isolated and identified from this natural product. Many studies conducted on various propolis samples have shown that the main secondary metabolites are phenolic substances, especially flavonoids, belonging to different sub-classes such as flavanones, flavones, flavonols and dihydroflavonols, which constitute more than 50% of the propolis weight [4, 9, 42–44]. These compounds, generally present in the plant kingdom as glycosides, are mostly present in propolis as aglycones, probably due to the action of bees’enzymes (glucosidase) during harvesting and transport. Other phenolic compounds found abundantly in propolis are hydroquinones, caffeic acids and related esters and phenolic aldehydes [48]. Propolis has also proved to be a rich source of essential elements such as magnesium, nickel, calcium, iron, zinc, cesium, manganese, silver, copper, aluminum, vanadium, vitamins B, C and E [49], as well as amino acids [50]. In addition, in propolis, some non-phenolic substances belonging to different classes such as aliphatic acids, coumarins, aliphatic and aromatic hydrocarbons, terpenoids, steroids and isoprenylated benzophenones, have also been found. The chemical composition of propolis is closely related to the place of origin, the time of harvest and the plant source from which it derives, and it is for this reason it is difficult to achieve a univocal classification of the chemical substances and therapeutic properties of this natural product. Moreover, these remarkable qualitative-quantitative phytochemical differences related to its geographical distribution that diversify and characterize propolis, make it unique both from chemical and biological point of view. For example, Tunisian propolis is distinguished by the presence of characteristic methoxyylated flavonoids, such as quercetin 3,7,3 ‘-trimethyl ether and myricetin 3,7,4',5' tetramethylether from *Cistus* spp., Cistaceae, leaf exudates [51], while a study carried out on New Zealand propolis has shown that hydroflavonoids such as pinocembrin and pinobanksin and make up about 70% of the total flavonoids from *Populus nigra* L., Salicaceae, bud exudates [52].

**Fig. 2:**
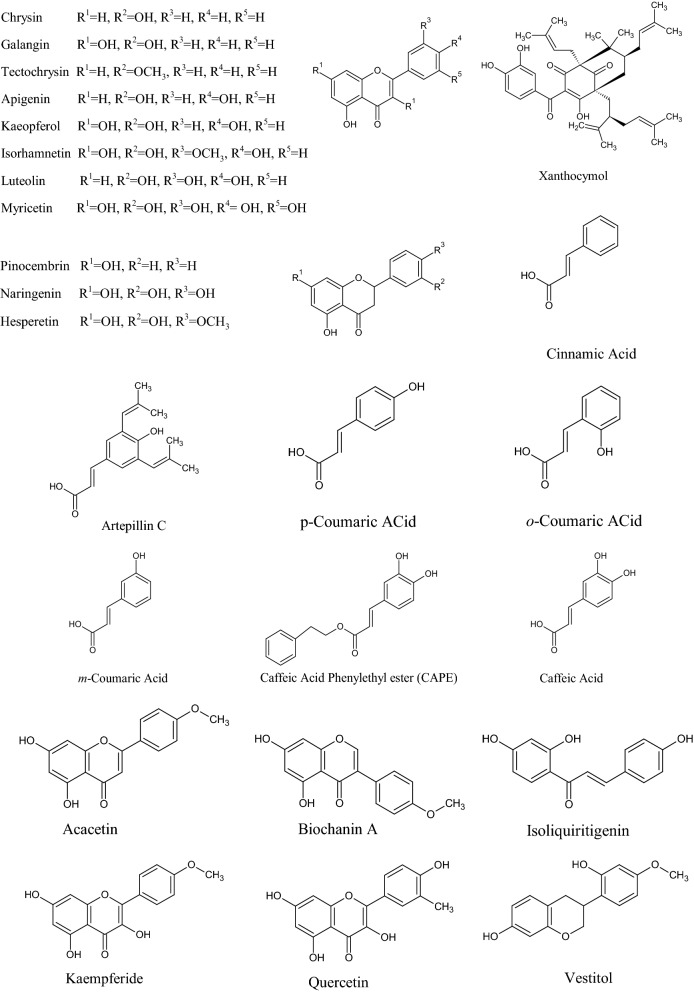

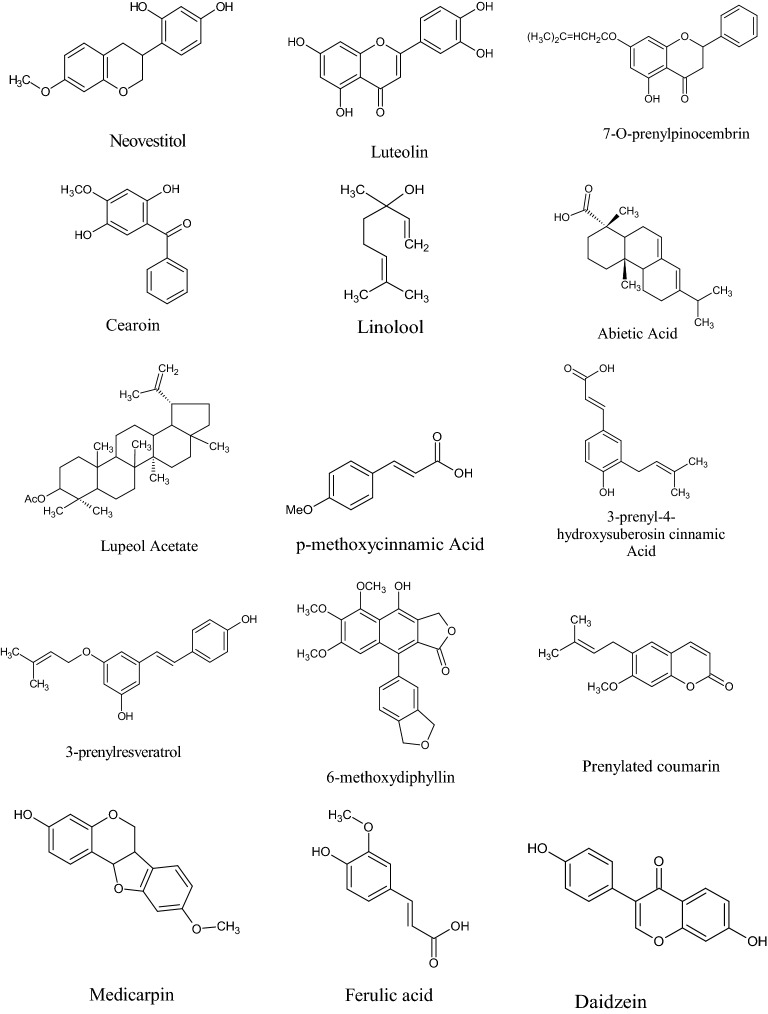

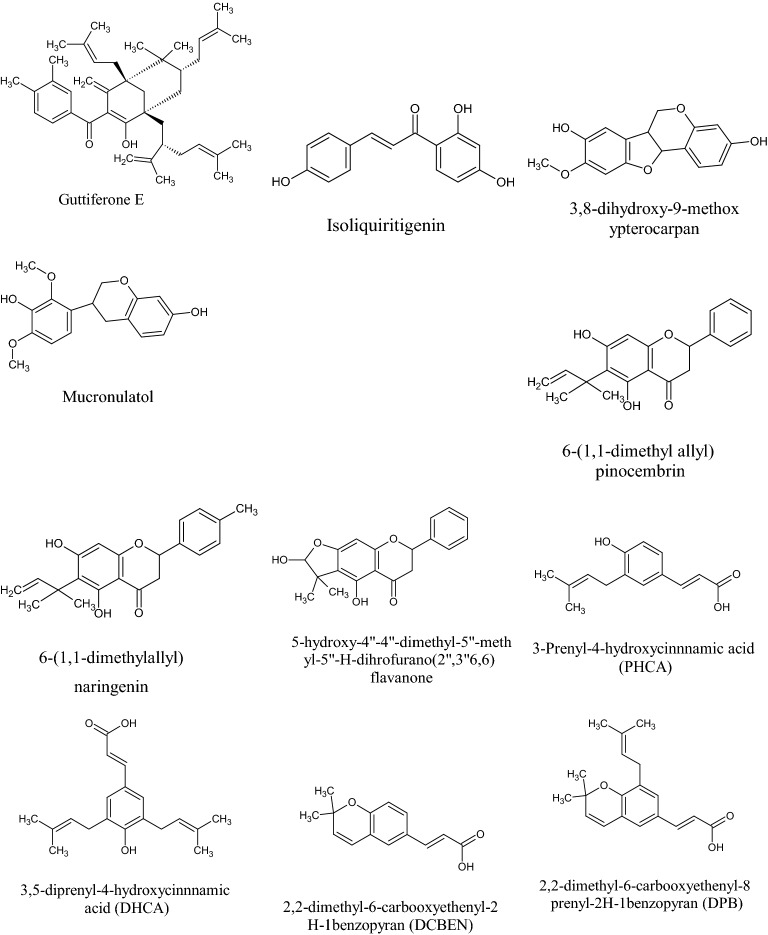

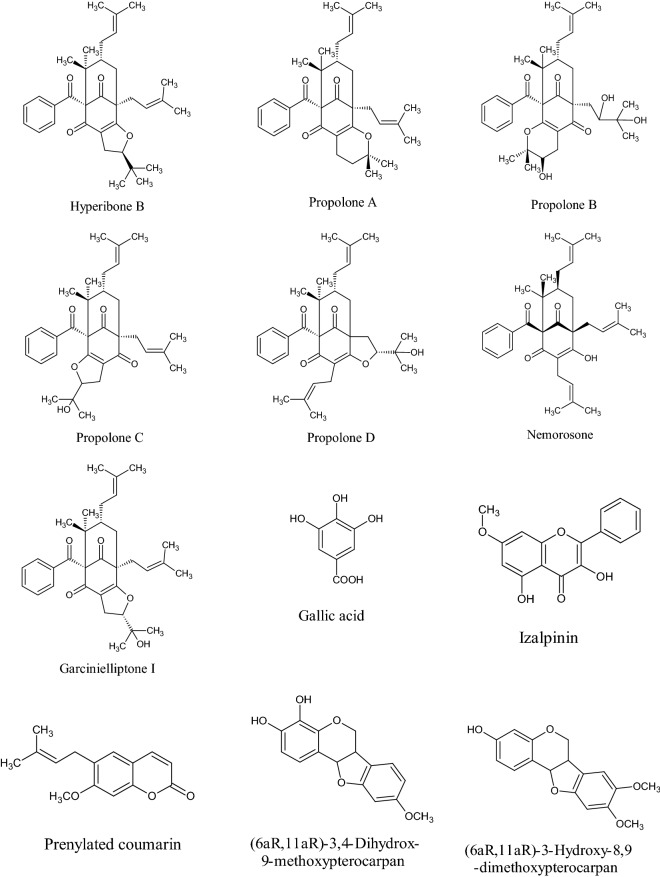

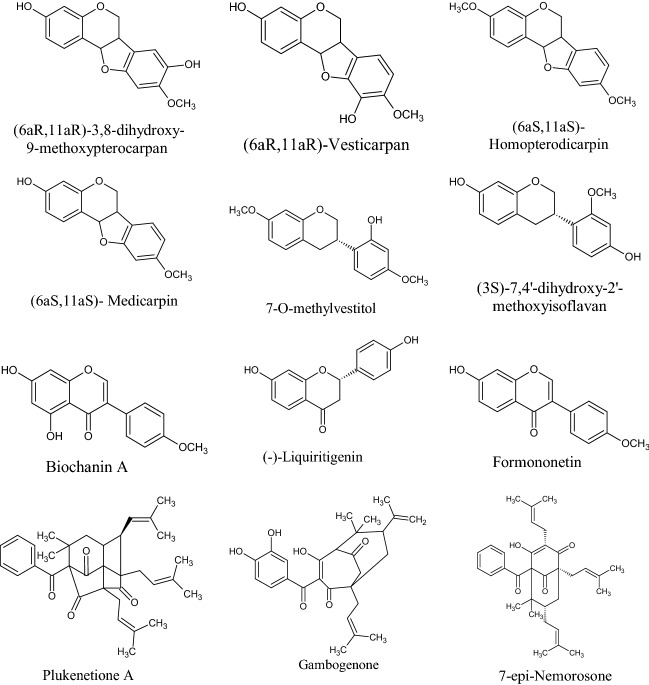

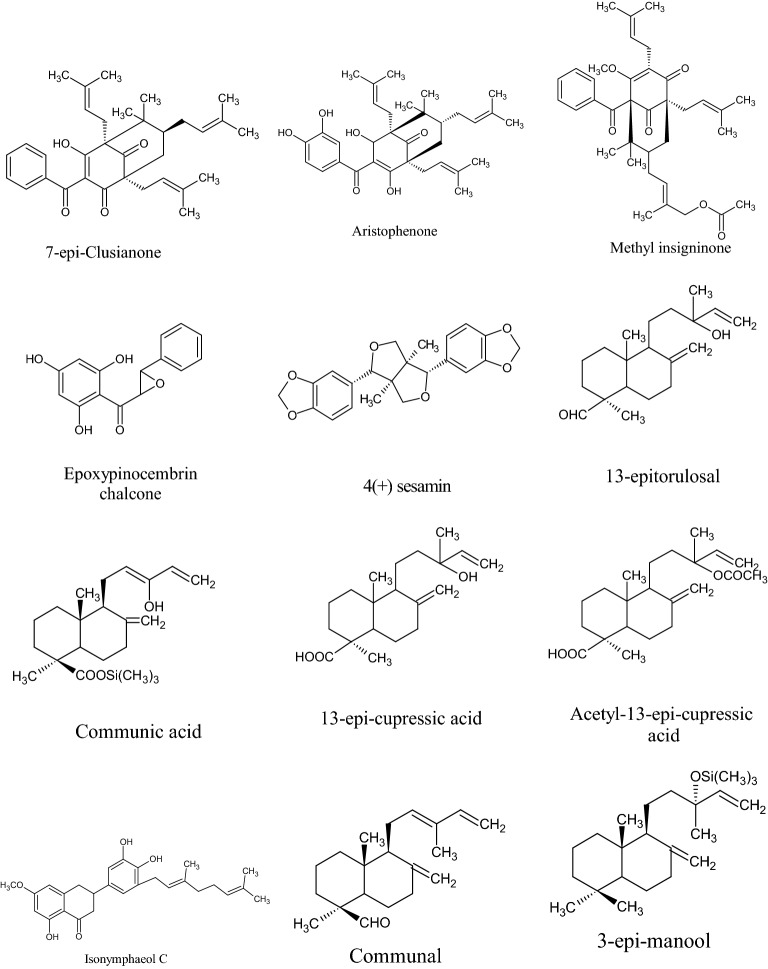

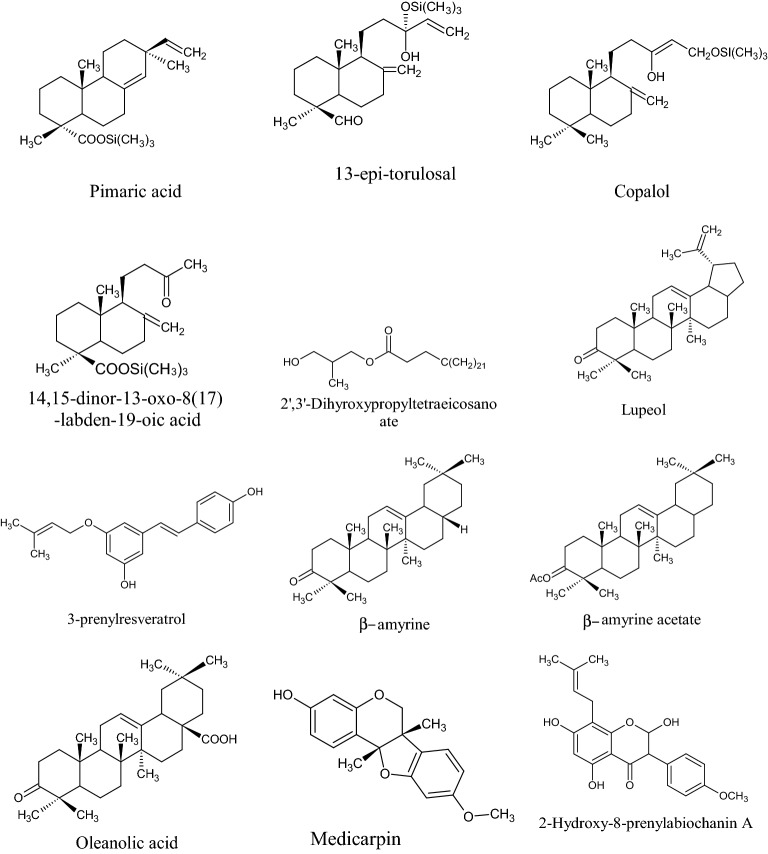

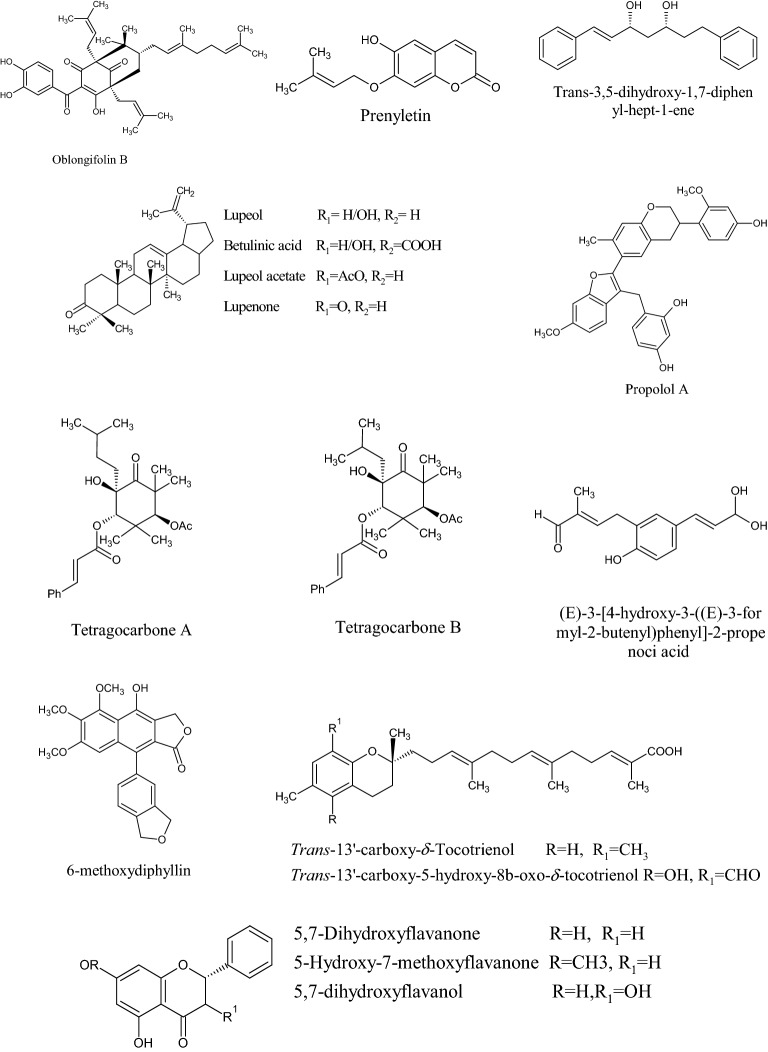

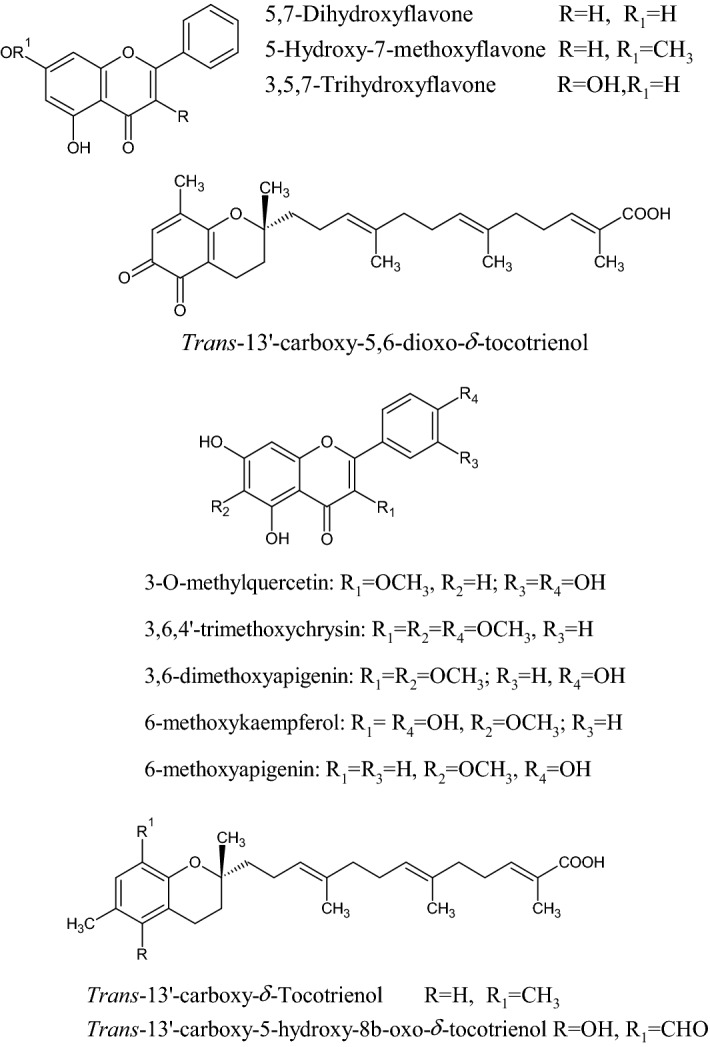
Isolated compounds from propolis

In the Uruguayan and the Chinese [53], however, these hydroflavonoids are present in less than 10%; while in Brazil they make up as much as 50% [49, 54, 55]. In the Chinese and Uruguayan varieties, the predominant flavonoids are substantially flavones and flavonols [53]. Extensive comparisons have been made on the chemical composition between propolis samples from Europe, South America and Asia [49], and from this point of view, it was possible to establish that European and Chinese propolis mainly abound in various species of flavonoids, phenolic acids and relative esters, while the predominant compounds of Brazilian propolis are terpenoids and prenylated derivatives of *p*-coumaric acid [49, 54, 55], such as artepillin C of Brazilian green propolis from the plant *Baccharis dracunculifolia* DC, Asteraceae, in southeastern and western-central Brazil [56, 57]. Therefore, there is a considerable difference between propolis originating in tropical areas (South America) with respect to those of temperate areas (Europe). Indeed, the first one is caractherized by substances with a hydroxycinnamic acid nucleus (C_6_–C_3_ backbone), whereas in the second one the flavonoid (C_6_-C_3_–C_6_ backbone) composition predominates [49]. In Fig. 2, the 2D structures of the main isolated compounds from propolis were depicted, whereas the main compounds isolated from African, American, Australian and Asian propolis were reported in Tables 2, 3, 4, 5, and 6.

**Table 2:** Compounds isolated from African propolis

Country	Compound	Class	References
Cameroon	Lupenone, α-amyrin, β-amyrin, methyl-3β,27-dihydroxycycloart-24-en-26-oate, oleanolic acid, β-amyrin acetate, lupeol, betulinic acid, lupeol acetate, ψ-taraxasterol-acetate, taraxasterol acetate, lanosterol, 3α-hydroxy-olean-12-en-30-ol, α-amyrone, β-acetoxy-amyrin, bacchara12,21dien3β-ol, betulinaldehyde and erythrodiol	Terpenoid	[405, 406]
	3-Undecylphenol, 3-tetradecylphenol, 3-pentadecylphenol, 3-hexadecylphenol, 3-heptadecylphenol, 3-nonadecylphenol, 3-((100 z)-pentadecenyl)-phenol, 3-((120 z)-pentadecenyl)-phenol, 3-((80 z)-heptadecenyl)-phenol, 3-((120 z)-heptadecenyl)-phenol, 3-((140 z)-heptadecenyl)-phenol, 3-((130 z)-nonadecenyl)-phenol, 3-((140 z)-nonadecenyl)-phenol, 5-pentadecylresorcinol, 5-hexadecylresorcinol, 5-heptadecylresorcinol, 5-((100 z)-pentadecenyl)-resorcinol, 5-((80 z)-heptadecenyl)-resorcinol, 5-((110 z)-heptadecenyl)-resorcinol, 5-((120 z)-heptadecenyl)-resorcinol, 5-((140 z)-hheptadecenyl)-resorcinol, 5-((140 z)-nonadecenyl)-resorcinol	Phenolic lipids	[407]
Cameroon	α-Terpineol and 1,8-terpineol	Monoterpene alcohols	[406]
	Ethyl palmitate	Fatty acid ester	
	Lonchocarpol a, 6,8-diprenyl-aromadendrin and lespedezaflavanone C	Diprenyl-flavonoids	
Congo	Lupenone, β-amyrin and lupeol	Triterpenes	
	lonchocarpol A and 6,8-diprenyl-eriodictyol	Diprenyl-flavonoids	
Cameroon	10-*O*-Eicosanylglycerol, deperoxidized derivative of plukenetione C	Miscellanous	[405, 408]
Egyptian	2,3-dihydroxy-4-methyl-octanoic acid hexacosanoic acid; 3,4- dimethoxy-cinnamic acid; 3-methyl-3-butenyl-trans-caffeate ester, tectochrysin 5-hydroxy-7-methoxy isoflavone		[409]
Egyptian	Isonymphaeol C	Prenylated flavonoid	[409]
Libya/Nigeria/cameroon	Cycloartanol, mangiferolic acid, mangiferonic acid, ambolic acid, 27-hydroxymangiferonic acid	Cycloartane triterpene	[405]
Nigeria	Ambonic acid		[410]
Libya	13-Epitorulosol, acetylisocupressic acid, agathadiol, isocupressic acid, isoagatholal	Diterpene	[411]
Cameroon	2-Hydroxy-8-prenylbiochanin A	Flavonoid	
Libya	Taxifolin-3-acetyl-4′methyl ether		
Nigeria	3,8-Dihydroxy-9-methoxy-pterocarpan, astrapterocarpan, vesticarpan, vestitol, broussonin B, calycosin, liquiritigenin, pinocembrin, isosativan, 2′-hydroxy-7,4′-dimethoxyisoflavan, medicarpin	Flavonoid	[411]
Algeria	Pectolinarigenin; 6,7-dihydroxy-7,4′-dimethoxyflavone (ladanein), acacetin, quercetin, 3-*O*-methyl-quercetin, kaempferol, chrysin, tectochrysin, galangin, myricetin-3,7,40,50-tetramethyl ether, apigenin, pectolinarigenin, pilosin, ladanein, pinocembrin, pinobanksin, pinobanksin-3-acetate, pinobanksin-3-(E)-caffeate, pinostrobin, genistein,	Flavonoid	[412, 413]
	*(E)-*resveratrol, tyrosol	Miscellanous	[413]
	pimaric acid, totarol, 18-hydroxy-cis-clerodan-3-en-15-oic acid, cistadiol, isoagathotal, imbricatoloic acid, cupressic acid, isocupressic acid, torulosol, agathadiol, torulosal	Terpenoids	[412, 413]
	caffeic acid, prenyl caffeate, methyl caffeate, isopentyl caffeate, 2-methyl-2-butenyl (E)-caffeate, 3-methyl-3-butenyl-(E)-caffeate, phenethyl-(E)-caffeate (CAPE), *p*-coumaric acid, *p*-coumaric acid methyl ester, cinnamic acid (isoferulic acid, caftaric acid, caftaric acid methyl ester, ( +)-chicoric acid, ( +)-chicoric acid methyl ester,	Phenylpropanoids	[412–414]
Egypt	Isoferulic acid	Phenylpropanoids	[415]
	Chrysin, tectochrysin, galangin, pinocembrin, pinostrobin, quercetin-3,7-di-o-methyl ether, 3-methoxy-5,7,40 –trihydroxyflavone, 3,30 -dimethoxy-5,7,40 –trihydroxyflavone, izalpinin, isonymphaeol C, isonymphaeol B, isonymphaeol D, nymphaeol B,	Flavonoids	[409, 415]
	3β-Cycloartenol, 3β-cycloartenol-26-oic acid, 3α-cycloartenol-26-oic acid, β-amyrin acetate,	Terpenoids	[416]
Nigeria	Macarangin, 8-prenylnaringenin, pinocembrin, nymphaeol b, liquiritigenin, calycosin, (3S)-vestitol	Flavonoids	[417]
	Ambonic acid, mangiferonic acid, α-amyrin	Terpenoids	[405, 410]
	Medicarpin, riverinol, gerontoxanthone h, 6-deoxy-γ-mangostin, 1,7-dihydroxy-3-o-(3-methylbut-2-enyl)-8-(3-methylbut-2-enyl) xanthone	Miscellanous	[410]
	8-prenylnaringenin, 6-prenylnaringenin, propolin d, macarangin	Prenylated flavonoid	[405, 410]
	Gerontoxanthone H, 6-deoxy-γ-mangostin; 1,7-dihydro-3-*o*-(3-methylbut-2-enyl)-8(3-methylbut-2-enyl) xanthone	Xanthone	
Libya	13-Epitorulosal, acetyl-13-epi-cupressic acid, 13-epi-cupressic acid	Diterpenes	[418]
	Sesamin	Lignin	
	Taxifolin-3-acetyl-4′-methyl ether	Flavanonol	
	13-Epitorulosolol	Diterpene	
	Demethylpiperitol, 5′-methoxypiperitol	Lignan	
	Cycloartanol, mangiferolic acid, mangiferonic acid, ambolic acid, 27-hydroxymangiferonic acid	Cycloartane triterpene	
	Cardol	Resorcinol	
	Agathadiol, isocupressic acid, isoagatholal, acetylisocupressic acid	Diterpene	
	bilobol		
Libya	Demethylpiperitol, 5′-methoxypiperitol	Lignan	[411]
Nigeria	Riverinol	Benzofuran	
Cameroon	Triacontyl ρ-coumarate	Coumarin	
	Arachic/arachidic acid ethyl ester (PEN_4_)	Alkylphenol	
Libya/Cameroon	Cardol	Alkylresorcinol	
Cameroon	1′-*O*-Eicosanyl glycerol	Acylglycerol	[405]
Nigeria	Oleic acid; propyl stearate	Fatty acid and ester	[410]
Cameroon	Hexatriacontanoic acid, 2′,3′-dihydroxypropyltetraeicosanoate	Fatty acid	[411]
Zambian and Tanzanian	6(1,1, dimethyl allyl) pinocembrin; 6(1,1, dimethyl allyl) naringenin; 5-hydroxy-4″,4″-dimethyl-5″-methyl-5″-*H*-dihydrofuranol [2″,3″,6,7] flavanone	Flavanone	[419]
Mediterranean	Copalol; 3-epi-manool; communal; 14,15-dinor-13-oxo-8(17)-labden-19-oic acid; pimaric acid; 13-epi-torulosal; communic acid;	Diterpene	[389]
Cameroon	2-Hydroxy-8-prenylabiochanin A, 2’,3’-dihydroxypropyltetraeicosanoate, β-amyrine, oleanolic acid, β-amyrine acetate, lupeol, betulinic acid, lupeol acetate, lupenone	Isoflavonol	[219]
Kenya	Tetrahydrojusticidin B, phyllamyricin C, and 6-methoxydiphyllin	Arylnaphtalene lignan	[376]
Kenya	Macarangin, schweinfurthin A and B	Geranylated flavonol (geranylstilbenes)	[376]
Kenya	Phyllamyricin C, tetrahydrojusticidin B, 6-methoxydiphyllin	Micellanous	[376]
Cameroon	(E)-5-(2-(8-hydroxy-2-methyl-2-(4-methylpent-3-en-1-yl)-2H-chromen-6-yl) vinyl)-2-(3-methylbut-2-en-1-yl) benzene-1,3-diol; 5-((E)-3,5-dihydroxystyryl)-3- ((E)-3,7-dimethylocta-2,6-dien-1-yl) benzene-1,2-diol;	Prenylated stilbenes	[408]

**Table 3:** Compounds isolated from American propolis

Country	Compound	Class	References
Brazil	β-Amyrin, glutinol	Triterpenoid	[420]
Bolivia	Cycloart-24-en-3β-ol, cycloart-24-en-3β,26-diol,		
	24(*E*)-Cycloart-24-en-26-ol-3-one, cycloart-24-en-3-one, lupeol, cycloartenone	Cycloartane triterpene	[421]
Brazil	Liquiritigenin, isoliquiritigenin, formononetin, vestitol, neovestitol, medicarpin, 7-*O*-neovestitol, 3-*O*-methylquercetin, 3,6,4′-trimethoxychrysin, 3,6-dimethoxyapigenin, 6-methoxykaempferol, 6-methoxyapigenin, 5,7-dihydroxy-2-(3,4-dihydroxy)-4 h-chromen-4-one, dihydrokaempferide	Flavonoid	[103, 422, 423]
	3-O-Methylquercetin, 3,6,4'-trimethoxychrysin, 3,6-dimethoxyapigenin 6-methoxyapigenin, 6-methoxykaempferol	Flavones	[103]
	3-Prenyl-4-hydroxycinnamic acid, 3,5-diprenyl-4-hydroxycinnamic acid, 2,2-dimethyl-6-carbooxyethyl-2-H-1-benzopyran, 2,2-dimethyl-6-carbooxyethyl-2–8-prenyl-2H-1-benzopyran	Polyphenol	[397]
	nemorosone, gambogenone, 7-epi-Nemorosone, 7-epi-clusianone, methylinsigninone, aristophenone, hyperibone B	Polyisoprenylated benzophenones	[95, 424]
			[425]
	Propolonones A, propolonones B, propolonones C, propolol A	Flavonoid derived dimer	[102]
	(*E*)-3-[4-Hydroxy-3-((*E*)-3-formyl-2-butenyl)phenyl]-2- propenoic acid, 3,4-dihydroxy-5-prenyl-(*E*)-cinnamic acid, capillartemisin A, 2,2-dimethylchromene-6-(*E*)-propenoic acid	Cinnamic acid derivative	[423]
Chile	5,7-Dihydroxyflavanone, 5-hydroxy-7-methoxyflavanone, 5,7-dihydroxyflavanol, 5,7-dihydroxyflavone, 5,hydroxy-7-methxyflavone, 3,5,7-trihydroxyflavone	Flavonoid	[426]
Colombian	Trans-13’-carboxy-5-hydroxy-8b-oxo-δ-tocotrienol, trans-13’-carboxy-5,6-dioxo-δ-tocotrienol, trans-13’-carboxy-δ-tocotrienol	d-Tocotrienol	[427]
Central Chilean Matorral	Prenyletin, trans-3,5-dihydroxy-1,7-diphenyl-hept-1-ene, acacetin, izalpinin, kaempferide	Phenolic	[428]
Cuba/ Brazil	Propolone A, propolone B, propolone C, propolone D	Polyisoprenylated Benzophenones/ Flavonoid	[102, 424, 429]
Cuba	Gallic acid, isoliquiritigenin, liquiritigenin, formononetin, biochanin A, (3S)-7,4’-dihydroxy-2’-methoxyisoflavan, 7-*O*-methylvestitol, (6aS,11aS)-medicarpin,(6aS,11aS)-homopterodicarpin, (6aR,11aR)-vesticarpin,(6aR,11aR)-3,8-dihydroxy-9-methoxypterocarpan, (6aR,11aR)-3-hydroxy-8,9 dimethoxypterocarpan, (6aR,11aR)-3,4-dihydroxy-9-methoxypterocarpan	Isoflavonoids	[378]
	Plukenetione A	Polyprenylated acylphloroglucinol	[430]
Mexican	Pinocenbrin, pinobanksin chrysin, galangin-5-methylether, isorhamnetin, pinobanksin-5-methylether, alpinetin, alpinone, pinostrobin, kaempferide	Flavonoid	[431]
Ecuador	Naringenin, Sakuranetin, Eupatolitin, Rhamnazin		[432]
Chile	Pinocembrin, chrysin		[433]
Bolivia	Kaempferol 3-methyl ether, kaempferol 7-*O*-methyl ether		[421]
Brazil	2-Phenoxychromone	Benzopyran derivative	[434]
Bolivia	Cinnamic acid	Phenyl propanoid	[421]
	3-Prenyl-*p*-coumaric acid (drupanin)	Coumarin	
	Benzyl benzoate	Benzyl ester	
Brazil	Guttiferone E, oblongifolin B	Polyprenylated benzophenone	[434]
	2-Phenoxychromone	Flavones	[103]
Chile	(*E*)-3-Hydroxy-1,7-diphenylhept-1-ene-5-acetate, (*E*)-5-hydroxy-1,7-diphenylhept-1-ene-3-acetate	Diarylheptanoid	[435]

**Table 4:** Compounds isolated from Australian propolis

Country	Compound	Class	References
Pitcairn Island	3-oxo-cycloart-24*e*-en-21,26-diol-21,26-diacetate, 3-oxo-cycloart-24*e*-en-21,26-diol, 3-oxo-cycloart-24e-en-21,26-diol-21-acetate, 3-oxo-cycloart-24e-en-21,26-diol-26-acetate, 3-oxo-cycloart-24-en-26-al	Triterpenoid	[436, 437]
Australia	7,8,18-Trihydroxyserrulat-14-ene, 5,18-epoxyserrulat-14-en-7,8-dione, (18*RS*)-5,18-epoxyserrulat-14-en-8,18-diol	Diterpene	[12]
Pitcairn Island	Abietinal	Diterpene	[436, 437]
Fiji Island	Glyasperin	Flavonoid	[438]
Kangaroo Island	(*E*)-4-(3-Methyl-2-buten-1-yl)-3,4′,5-trihydroxy-3′-methoxystilbene, (*E*)-2-(3-methyl-2-buten-1-yl)-3,4′,5-trihydroxystilbene (2-prenylresveratrol), (*E*)-2,4-bis(3-methyl-2-buten-1-yl)-3,3′,4′,5-tetrahydroxystilbene (*E*)-2-(3-methyl-2-buten-1-yl)-3-(3-methyl-2-butenyloxy)-3′,4′,5-trihydroxystilbene, (*E*)-2,6-bis(3-methyl-2-buten-1-yl)-3,3′,5,5′-tetrahydroxystilbene (*E*)-2,6-bis-(3-methyl-2-buten-1-yl)-3,4′,5-trihydroxy-3′-methoxystilbene	Stilbene	[398, 439, 440]
Fiji Islands	Solomonin B, solomonin C	Stilbene	[438]
	Glyasperin A, kumatakenin, macarangin, mangiferolic acid	Flavonid

**Table 5:** Compound isolated from Asian propolis

Country	Compounds	Class	Refs.
Indonesia/Vietnam	Mangiferolic acid, cycloartenol, mangferonic acid, ambonic acid, ambolic acid	Cycloartane triterpenoid	[238, 441]
	Cardol	Alkyresorcinol	[138]
Vietnam	23-Hydroxyisomangiferolic acid B, 23-hydroxyisomangiferolic acid A, 27-acetoxymangoferolic acid, (5R,8 S,9 S,10 R,13 R,14 S,17 R,20 R)-27-methoxycarbonyloxymangiferonic acid, 27-acetoxymangoferonic acid, lanosterol, cycloartenone, mangiferonic acid, 23-hydroxymangiferonic acid, 27-hydroxymangiferonic acid, (23E)-27-nor-3β-hydroxycycloart-23-en-25-one, (24E)-3βhydroxycycloart-24-en-26-al, 27-hydroxyisomangiferolic acid	Cycloartane triterpenoid	[442]
Thailand/Vietnam	3-*O*-Acetyl ursolic acid, ocotillone I, ocotillone II, ursolic aldehyde, oleanolic aldehyde, dipterocarpol, cabralealactone, isocabralealactone	Triterpenoid	[441, 443]
Thailand	Methylpinoresinol	Lignan	[443]
Malaysia	20-Hydroxy-24-dammaren-3-one, β-panasinsene	Sesquiterpene	
Thailand/Vietnam	α-Mangostin, γ-mangostin, cochinchinone t, β-mangostin, gartanin, 8-deoxygartanin, 9-hydroxycalabaxanthone, mangostanol, mangostanin, garcinone B	Prenylated xanthone	[441, 443]
Vietnam	Hydroxyhopanone	Micellanuous	[441]
	(13*E*,17*E*)-polypoda-7,13,17,21-tetraen-3β-ol		
	Lisofurvin,	Dihydrochromene	[441]
	Dihydrobrasixanthone B, cochichinone A, cochichinone I, cochichinone J, cratoxylumxanthone B, α-mangostanin, pruniflorone S, 2-hydroxyl-6-(14'*Z* -nonadecenyl) benzoic acid	Xanthone	
		Alkenylphenol	[444]

**Table 6:** In vitro antimicrobial effects of some propolis extracts, doses, and studied microorganisms

Country/Region	Solvent	Dose/Concentration	Microorganism	Effect	References
Tunisia	Ethanol	10 μl	*Aspergillus flavus, Aspergillus niger, Streptococcus mutans, Penicillium nordicum, Penicillium expansium, Penicillium commune, Lactobacillus plantarum, Escherichia coli*	Growth inhibition	[83]
Chile	Ethanol	MIC, 256 to 1024 μg/ml	*Helicobacter pylori* ATCC 43,504, *Helicobacter pylori* 84C	Growth inhibition	[37]
Brazil	Ethanol	MIC 7.44 to 29.76 mg/ml	*Streptococcus mutans* ATCC 25,175, *Streptococcus sanguinis* ATCC 10,566, *Streptococcus salivarius* ATCC 7073, *Lactobacillus casei* ATCC 393	Growth inhibition	[93]
-	-	200 μl	*Streptococcus mutans* ATCC 25,175, *Streptococcus sanguinis* ATCC 10,566, *Candida albicans* ATCC 1023	Growth inhibition	[92]
Mexico	Ethanol	MIC 1.62 to 2.50 μg/ml	*Candida albicans* ATCC 10,231	Growth inhibition	[46]
Turkey	Ethanol–water (8:2)	0.25, 0.50, 1 mg/well	*Streptococcus sanguinis, Streptococcus pyogenes, Streptococcus mutans, Candida albicans*	Growth inhibition	[47]
Algeria	Ethanol–water	500 μg/ml	*Shewanellaputrefaciens, Photobacterium damselae, Vibrio harveyi*	Growth inhibition	[87]
India	Chloroform, ethanol, acetone	5,10,15 mg/ml	*Pseudomonas flurorescens, Pseudomonas citrea, Pseudomonas aeruginosa,* *Staphylococcus aureus, Escherichia coli, Vibrio cholera, Vibrio parahaemlyticus, Vibrio harveyi, Vibrio fischeri, Bacilliussubstillus*	Growth inhibition	[76]
Brazil	Ethanol–Water	1. 64 to ≥ 1024 μg/ml	*Pseudomonas aeruginosa, Staphylococcus aureus, Escherichia coli*	Growth inhibition	[445]
Russia	Ethanol	2 μg/ml	*Staphylococcus aureus, Escherichia coli*	Anti-biofilm	[35]
Romania	Ethanol	10 mg/ml	*Paenibacillus larvae*	Growth inhibition	[88]
Argentina	Ethanol	MIC, 4.7 to 152 g GAE/ml	*Erwinia carotovorasppcarotovora* CECT 225, *Pseudomonas syringaepvar tomato* CECT 126, *Pseudomonas corrugata* CECT 124, *Xanthomonas campestris pvarvesicatoria* CECT 792	Growth inhibition	[86]
Korea	Ethanol	0.036 to 2.3 mg/ml	*Bacillius cereus, Listeria monocytogenes, Staphylococcus aureus, Pseudomonas fluorescence*	Growth inhibition	[84]
Brazil	Ethanol	0.69 to 42.8 mg/ml	*Staphylococcus aureus, Staphylococcus intermedius, Malassezia pachydermatis*	Growth inhibition	[251]
Turkey	Ethanol	MIC, 4 to 512 mg/ml	*Peptostreptococcusanaerobius, Peptostreptococcus micros, Lactobacillus acidophilus, Actinomyces naeslundii, Prevotellaoralis, Prevotellamelaninogenica, Porphyromonasgingivalis, Fusobacterium nucleatum, Veillonellaparvula*	Growth inhibition	[38]
Brazil	Ethanol	0.4 to 0.5 mg	*Staphylococcus aureus* 209, *Escherichia coli, Candida albicans* 562	Growth inhibition	[446]

Propolis from Egypt reported to possess constituent of *P. nigra* and esters of caffeic acid and long-chain fatty alcohols including tetradecanol, hexadecanol and dodecanol [9]. Cuban propolis is mainly from *Clusia rosea* Jacq. and contains polyisoprenylated benzophenone which is distinct from both European and Brazilian propolis [2, 9]. Birch propolis from Russia contained flavonols and flavones from *Betula pendula* Roth. Anjum et al. [4].

## Analytical techniques

Each propolis type is characterized by the specific proportion of the dominant plant material. Various chromatographic techniques were used for evaluation of the botanical origin of propolis samples such as high performance thin-layer chromatography [58], high-performance liquid chromatography (HPLC) [59–61], gas chromatography-mass spectrometry (GC–MS) [62], capillary electrophoresis [63], microscopic techniques [64]. Furthermore, structure elucidation techniques such as direct analysis in real time (DART) mass spectrometry [52], nuclear magnetic resonance (NMR) [65], and infrared spectroscopy (IR) [66] have been applied to screen compositional differences between propolis samples supplied from various origins.

Using high-performance thin-layer chromatographic (HPTLC) fingerprinting analyses to explore the chemical composition of propolis, studies have confirmed the existence of two different subtypes of European propolis, as orange and blue types (O-type and B-type), originating from *P. nigra* and *Populus tremulas* L., respectively. Green type (G-type) propolis is distinguished by its mixture of light orange, dark green, and blue bands. O-type propolis is characterized by quercetin, while B-type corresponds mostly to galangin, caffeic acid, feruloyl, and *p*-coumaroyl derivatives. G-type corresponds to apigenin or naringenin. For instances, HPTLC profiles of plant resins as potential botanical sources of propolis, were compared with profiles of Turkish propolis extracts [58]. Based on phenolics fingerprint, O-type of Turkish propolis showed similarity with *P. nigra* buds, while B-type has similar HPTLC pattern with *P. tremula* bud.

Recently, a reversed-phase HPLC-ESI-TOF–MS analysis of an ethyl acetate extract of propolis prepared from *Apis mellifera* beehives using liquid–liquid extraction was described. Two flavonoids, galangin and apigenin, were identified as the major constituents present in this bee glue, which were responsabible for the inhibitory activity in head and neck squamous cell carcinoma on the key enzymes matrix metalloprotease (MMP)-2 and MMP-9 that destroyed the basement membrane and degrade the extracellular matrix, leading to tumor invasion Niyomtham et al. [59].

## Pharmacological activities

### Antioxidant activity

The screening of the biological activity of bee products has attracted considerable attention due to their health benefits. Bee products including propolis, are considered to be a potential source of naturally occurring antioxidant and antimicrobials [67–72].

Several antioxidant tests such as 2,2-diphenyl-1-picrylhydrazyl (DPPH), trolox equivalent antioxidant capacity (TEAC), ferric reducing antioxidant power (FRAP), iron-chelating activity (ferrozine assay), cupric ions reducing antioxidant power (CUPRAC), hydroxyl radical scavenging ability, lipid peroxidation inhibition, N,N-dimethyl-*p*-phenylenediamine (DMPD), O_2_ scavenging ability, H_2_O_2_ scavenging ability, β-carotene bleaching, superoxide anion radical scavenging ability have been used to evaluate the in vitro antioxidant activity of propolis [43, 67, 73]. These tests have different mechanisms, reaction environments, principles, charged radicals, and use difference reference compounds. Therefore, the antioxidant ability of natural compounds is generally evaluated with at least two or more antioxidant methods. Besides this, the phenolic content and composition of natural products is determined and correlated with the antioxidant capability in order to validate the obtained results [42, 74].

The extraction method greatly influences the type and quantity of bioactive compounds, because their solubility also depends on the solvent type and polarity [75, 76]. The antioxidant efficacy of propolis extracts (ethanol and water) as well as that of its fractions has been already reported in several studies [7, 43, 67, 77]. Ahn et al. [67] evaluated the antioxidant activity of ethanol extracts of propolis from various Chinese areas using the DPPH, TEAC and β-carotene bleaching assays. Chinese propolis extracts showed strong antioxidant activity with a positive correlation with the high total phenols content, such as flavonoids and phenolic acid esters, identified by HPLC coupled with a photodiode array (PDA) and mass spectrometry (MS) detection [60]. There are many other reports concerning the antioxidant potential of ethanol extract of propolis [44, 78, 79]. In addition, there are also some reports about the antioxidant capacity of water extracts of propolis [7]. The antioxidant activity of a water extract of propolis from Brazil was evaluated using the superoxide scavenging ability assay, the DPPH assay, and the hydroxyl radical scavenging abilit assay. The extract showed the best free-radical scavenging ability against superoxide anion radical and hydroxyl radical at 50 and 100 µg/ml, respectively. Similarly, in the study of Gülçin et al. [43], who investigated the in vitro antioxidant activity of a water extract of propolis from Erzurum province of Turkey by using FRAP, CUPRAC, iron-chelating activity, H_2_O_2_ and O_2_ sscavenging ability as well as DPPH and TEAC assays, the water extract showed an interesting antioxidant profile. The aqueous extract was analyzed by HPLC and LC–MS/MS reporting a large number of antioxidative compounds such as caffeic acid, ferulic acid, syringic acid, ellagic acid, quercetin, α-tocopherol, pyrogallol, *p*-hydroxy benzoic acid, vanillin, *p*-coumaric acid, gallic acid, and ascorbic acid [43]. In the study conducted by Aldemir et al. [80], the effect of different solvents (ethanol, water and polyethylene glycol) on the antioxidant activity of propolis was determined using TEAC and FRAP methods. However, they did not find any statistically significant differences in the antioxidant activity between the different propolis extracts. In another study, propolis extracts from different regions of Korea were evaluated and compared to extracts obtained from Brazilian propolis [79]. Korea propolis extracts, which contained the highest total phenols and flavonoids content, showed the highest antioxidant activity in the linoleic peroxidation and DPPH assays. Flavonoids present in propolis play a crucial role in photoaging and psoriasis [81].

Hochheim et al. [77] examined the antioxidant activity of methanol, water, 70% ethanol, dichloromethane, ethyl acetate and butanol extract fractions in native Brazilian bee *Melipona quadrifasciata* propolis. The authors found that the aqueous extract showed the best antioxidant potential, followed by methanol extract. Moreover, the methanol extract showed the highest radical scavenging capacity in the DPPH assay with an IC_50_ value of 151.37 ± 7.9 μg/ml followed by ethyl acetate (IC_50_ = 311.47 ± 8.2 μg/ml), dichloromethane (IC_50_ = 489.8 ± 0.0 μg/ml), and butanol (IC_50_ = 588.8 ± 0.0 μg/ml) fractions. In addition, crude extracts and fractions showed different activity in the lipid peroxidation assay. Ethyl acetate fraction [64.93 ± 0.6 mg gallic acid equivalent (GAE)/g], showed the highest total phenols content followed by methanol extract (57.53 ± 0.2 mg GAE/g), aqueous extract (11.96 ± 1.5 mg GAE/g), dichloromethane fraction (8.10 ± 0.3 mg GAE/g), hydroalcoholic extract (4.87 ± 0.2 mg GAE/g), and was not observed in butanol fraction. On the contrary, the highest content of flavonoids was detected in the methanol extract [8.48 ± 0.3 mg quercetin equivalents (QUE)/g], followed by ethyl acetate fraction (4.23 ± 0.5 mg QUE/g), hydroalcoholic extract (3.99 ± 0.4 mg QUE/g), aqueous extract (3.67 ± 0.3 mg QUE/g), dichloromethane fraction (2.20 ± 0.3 mg QUE/g), and butanol fraction (0.78 ± 0.2 mg QUE/g).

In another study, twelve propolis samples from Greece, Greek island, and east Cyprus were analyzed for their antioxidant activity, as well as chemical composition [42]. The propolis ethanol extracts possessed potent DPPH scavenging activities from 0.33 to 1.11 mmol trolox equivalents (TE)/g extract and reducing power ability from 2.14 to 3.35 mmol ascorbic acid equivalent (AAE)/g. The antioxidant activity of propolis samples correlated with total phenol content as well as with the amount of the main identified compounds such as phenolic acids and their esters, anthraquinones, flavonoids and terpenes. Kumazawa et al. [74] performed an extensive study investigating the antioxidant potential of propolis collected from Argentina, Australia, Brazil, Bulgaria, Chile, China, Hungary, New Zealand, South Africa, Thailand, Ukraine, Uruguay, United States and Uzbekistan by using *β*-carotene bleaching and DPPH assays. HPLC–PDA and LC–MS analysis were also used to identify the major constituents of their ethanol extracts. All the propolis extracts exhibited significant free-radical scavenging activity against DPPH radical and inhibitory effects on *β*-carotene-bleaching, mainly attributable to the phenolic acids and flavonoids content. Regarding this, Lee et al. [68] isolated ten phenylpropanoic acid esters from ethanol extracts of Korean propolis. The antioxidant potential of these compounds, specifically caffeic acid phenethyl ester, caffeic acid benzyl ester, caffeic acid ethyl ester, ferulic acid benzyl ester, ferulic acid 3′,3′-dimethylallyl ester, 3,4-dimethoxycaffeic acid cinnamyl ester, coumaric acid cinnamyl ester, coumaric acid benzyl ester, cinnamic acid phenethyl ester, and cinnamic acid cinnamyl ester, were investigated by DPPH and ABTS assays. Among the tested compounds, caffeic acid phenethyl ester (CAPE), caffeic acid benzyl ester, and caffeic acid ethyl ester exhibited significant activity, ferulic acid benzyl ester, ferulic acid 3′,3′-dimethylallyl ester had moderate activity, and the other compounds did not show any activity. Russo et al. [71] reported that the antoxidant activity of the propolis extract is mainly ascribable to CAPE and galangin. In particular, the results showed that propolis extract (with and without CAPE), galangin and CAPE exhibited a dose-dependent antioxidant activity. Moreover, CAPE possessed a higher antioxidant activity with respect to galangin. On the contrary, regarding Portugal propolis, the collected in Bornes region showed the higest antioxidant capacity with the following IC_50_ values than the propolis from Fundão region [82]. The values varied between IC_50_ = 6.22 μg/ml (Bornes) and IC_50_ = 52.00 μg/ml (Fundão) in DPPH assay, and IC_50_ = 9.00 μg/ml (Bornes) and IC_50_ = 55.00 μg/ml (Fundão) in CUPRAC assay. Moreover, the results obtained showed a positive correlation with the phenolic content of propolis extracts.

Ozdal et al. [36] determined the antioxidant activity of propolis collected in 11 different geographical areas of Turkey by using DPPH and CUPRAC methods. Moreover, the bioaccessibility of propolis samples in terms of total antioxidant capacity of propolis extracts after in *vitro* simulated gastrointestinal digestion was evaluated. The antioxidant capacity of samples recorded by DPPH method ranging from 1370.6 ± 198 mg trolox equivalents (TE)/100 g and 6332.9 ± 114 mg TE/100 g, whereas in CUPRAC assay the antioxidant capacity ranging from 2461.6 ± 278 mg TE/100 g and 8580.3 ± 234 mg TE/100 g. Moreover, for most samples, the antioxidant capacity of propolis recorded by both methods increased after in *vitro* gastrointestinal digestion from oral to intestinal phase.

Despite the litle information available in literature, the content of bioactive compounds in propolis may be different in relation to the seasonal collection. The antioxidant ability of propolis collected in three different regions of Algarve in winter and spring (Serra do Algarve, Transição and Barrocal) as well as in South Portugal (Bornes and Fundão regions) were evaluated by using DPPH, TEAC, superoxide anion radical and metal-chelating activity assays [73]. Samples collected in winter showed the highest free-radical scavenging activity in DPPH assay (IC_50_ = 0.027–0.031 mg/ml) whereas samples collected in spring showed the highest metal-chelating activity. Moreover, propolis samples collected in Barrocal region possessed the higher radical scavenging capacity with respect to Serra do Algarve and Transição regions, without any correlation between seasonal variation and antioxidant assays.

Considering all these results, it is possible to affirm that the antioxidant activity of propolis is certainly correlated with its phytochemical profile, which can vary not only according to the pedoclimatic characteristics of the territory but also according to the harvest season. In any case, certainly, propolis has shown a strong antioxidant activity which supports its use as a replacement for many synthetic antioxidant agents such as butylatedhydroxyanisole, butylatedhydroxytoluene, and tertiary butyl hydroquinone.

### Antimicrobial activity

Propolis is also increasingly recognized for its antimicrobial (antibacterial/antifungal) properties. The antimicrobial efficiency of propolis mainly depends on its broad chemical diversity, extraction methods, applied concentration, collected season, region, and bee species. However, despite this variability, propolis maintained its functional properties [46, 75, 76, 83]. Because it contains many bioactive compounds, propolis possesses antibacterial and antifungal activity on a board range of microorganisms. Propolis extract was effective specifically on various foodborne pathogens [84], agricultural bacterial pathogens [85], phytopathogenic bacteria [86], marine opportunistic pathogenic bacteria [87] other than honeybee pathogens [88]. The antibacterial effects of propolis samples collected in different countries/regions around the word have been investigated by several studies grouped in Table 1. The antibacterial properties, either bactericidal or bacteriostatic, highlighted by in vitro studies, depends on concentration, treatment time, and bacterial mode of action [84], although the mechanism is often unknown. Propolis acts either by directly interacting with the microbial cells or by stimulating the immune system of the host cells [89]. The hypothesized mechanisms include the inhibition of bacterial adherence and division, decrease of bacterial mobility, disturbance of membrane potential, and increase in cell membrane permeability [47, 90]. These biological activities are strictly related to the presence of phenolic acids, flavonoids, terpene esters, chalcones, dihydrochalcones, terpenoids, aliphatic acids and esters, aromatic compounds, and metals [35]. As mentioned before, several factors play a key role on the propolis activity, being one of this the extraction method. For example, Gargouri et al. [83] demonstrated that ultrasonic extraction method increased notably the biological activity of the propolis extract with respect to the conventional liquid–liquid extraction method. Concerning the effect of the extraction method used, a study was conducted to evaluate different extraction solvents [75]. In particular, the antimicrobial efficacy of polyethylene glycol (PEG) 400, water, olive oil and the combination of them was investigated against *Staphylococcus aureus* ATCC 25,923, *Escherichia coli* 25,922, *Pseudomonas aeruginosa* ATCC 27,853, *Klebsiella pneumoniae* ATCC 33,499, *Bacillus cereus* ATCC 8035, *Candida albicans* ATCC 60,193. Among them, PEG/olive oil/water, PEG/water, showed similar or a higher significant inhibition in comparison with the hydroalcholic extract. From phytochemical point of view, caffeic acid, trans-*p*-cumaric acid, and galangin were determined in all extracts under investigations, whereas naringenin, kaempferol, and galangin were determined in PEG/olive oil/water and hydroalcholic extracts. Selvaraj et al. [76] demonstrated the effect of different solvents (ethanol, chloroform, and acetone) on the propolis antibacterial activity. The ethanol extract, in particular, was found to be the most effective against microbial growth.

Moreover, the propolis composition varies also between bee species, which can modify its antimicrobial capacity. Ramón-Sierra et al. [46] reported the antifungal activity of ethanol extracts of propolis obtained from two different bee species (*Apis mellifera* and *Melipona beecheii*) against *C. albicans* ATCC 10,231. According to their findings, *M. beecheii* propolis extract showed a lower MIC with respect to the *A. mellifera* propolis extract. Furthermore, Campos et al. [91] reported that propolis ethanol extract of *Melipona orbignyi* (Hymenoptera, Apidae), very rich in aromatic acids, phenolic compounds, alcohols, terpenes, and sugars, is effective against *Staphylococcus aureus* and *C. albicans*.

The antibacterial activity of the various propolis samples also varied according to the collection region. Ristivojević et al. [47] compared the antimicrobial activity of forty-eight propolis ethanol extract from various Turkey regions against *Streptococcus sanguinis, Streptococcus pyogenes, Streptococcus mutans, C. albicans* as well as examined the phytochemical composition and antioxidant activity. They concluded that the samples with strongest antimicrobial activity contain the highest total phenols, total flavonoid as well as cinnamic, ferulic, caffeic, chlorogenic, and *p*-coumaric acid content. Similarly, the antimicrobial activity of propolis extracts from different regions of Korea was evaluated and compared to Brazil propolis [79]. Korea and Brazil propolis extracts showed high antimicrobial activity against *S. aureus, Bacillus subtilis, Salmonella Typhimurium* and *C. Albicans*, whereas they did not show any activity against *P. aeruginosa*. The Yeosu and Cheorwon extracts showed higher antibacterial activity with respect to Yangpyeong, mainly related to their higher total phenols and flavonoid contents. Ambi et al. [35] hypothesized that metals may be involved in the antimicrobial properties of Russian propolis, by testing a crude ethanol extract and a metal-free ethanol extract against *S. aureus* and *E. coli* biofilm formation. The crude ethanol extract was more active than the metal-free ethanol extract on biofilm formation. However, at the same concentration, increasing the time exposure to 40 h, both extracts completely inhibit the *S. aureus* and *E. coli* biofilms. Several studies have been carried out also about the antimicrobial properties of propolis gainst oral cavities infections. Its efficacy was tested against several oral microorganisms such as *S. mutans* ATCC 25,175, *S. sanguinis* ATCC 10,566, *C. albicans* ATCC 1023 and *Lactobacillus casei* ATCC 393 [92, 93]. Propolis showed a good inhibitory activity on all oral pathogens, paving the way to an alternative application of this natura product to mouthwashes and toothpastes. From these studies, it can be highlighted that propolis antimicrobial potential varied according to its chemical composition. However, among extraction methods, solvent type and bee species, it may be concluded that floral diversity is the most responsible for the bioactivity of propolis.

### Anticancer activity

#### In vitro studies

##### Cytotoxic, antioxidant and apoptotic effects

Cytotoxic and apoptotic effects have been described for propolis and correlacted with its capacity to improve the antioxidant capacity in various disease states [94]. Castro et al. [95] isolated prenylated benzophenone and isoprenylated benzophenone from Brazilian propolis in order to evaluate their cytotoxic effects. They carried out an in vitro study using HeLa cervical cancer cells and they found that the both compounds exhibited good cytotoxic effects at 0.18 µM concentration [95]. A study by Vukovic et al. [96] reported antioxidant, cytotoxic and pro-apoptotic activities of flavonoids derived from propolis in colon (HCT-116) and breast (MDA-MB-231) cancer cell lines at 115.68 μM, 114.75 μM, 66.86 μM, and 50 μM concentrations [96]. Flavones from Mexican propolis at 4.6 μM concentration significantly triggered apoptosis-like morphological changes/cytotoxic effects in PANC-1 human pancreatic cell line [97]. Additionally, in a recent study, Rivero-Cruz et al. [98] revealed the cytotoxic activity of an ethanol extract of propolis (92.2 µg/ml) in glioblastoma cells cancer line. Another study by Alday et al. [99] evaluated the antiproliferative activity by using the flavoinds and derived esters isolated from propolis at various concentrations (49.1 µM; 52.1 µM; 67.0 µM; 49.9 µM; 51.3 µM; 76.6 µM) in B-cell lymphoma cell line. All the isolated compounds exhibited the antiproliferative activity through apoptotic induction [99]. The ethanolic extract of Turkish propolis at 76.9 ± 2.9 µg/ml concentration showed cytotoxic activity in human normal foreskin fibroblast cells at 31.7 ± 0.26 µg/ml concentration arresting the cell cycle in G1 phase, inducing endoplasmic reticulum stress, caspase activity, and apoptosis and reducing mitochondrial membrane potential in Human adenocarcinomic alveolar basal epithelial (A549) cancer cell line [100]. In another study, the ethanol extract of polish propolis displayed anti-proliferative and pro-apoptotic effect at 100 µM concentration on HCT 116 colon cancer and Me45 malignant melanoma cells [101].

In a recent study, Banzato et al. [102] isolated rare flavanoid dimers, propolones A−D, with a 3-{3-[(2- phenylbenzofuran-3-yl)methyl]phenyl}chromane skeleton, and propolonones A−C, with a 3-[3-(3-benzylbenzofuran-2-yl)phenyl]-chromane skeleton, as constituents of Brazilian red propolis by cytotoxicity-guided assays. Results of the chiral-phase HPLC analysis of these propolis dimers confirmed that they represented scalemic mixtures in which the (+)-enantiomers predominate. The bees that produce this type of red propolis feed on *Dalbergia ecastophyllum* (L.) Taub. Propolone B and propolonone A display significant cytotoxic activities against an ovarian cancer cell line expressing a multiple drug resistance phenotype when compared with doxorubicin. They reported that the isolated compounds at 19.1 ± 2.4 µM and 29.9 ± 3.4 µM concentration exhibited suitable anti-proliferative activity in ovarian cancer cell line [102].

Mitsui et al. [103] also isolated flavones from Brazilian propolis and performed an in vitro study to evaluate the activity of these isolated compounds. They reported that 3-methoxy-flavones, such as 3-*O*-methylquercetin (MCF-7: IC_50_ 16.2 μM), 3,6-dimethoxyapigenin (MCF-7: IC_50_ 31.0 μM), and 3,6,4’-trimethoxychrysin (MCF-7: IC_50_ 17.1 μM), exhibited anti-proliferative effect and inhibited cell growth in DLD-1 (human colorectal adenocarcinoma), MCF-7 (human breast cancer), and A549 (human lung cancer) cancer cell lines. Interestengly, the novel 5,7-dihydroxy-2-[3,4-dihydroxyphenoxy-4*H*-chromen-4-one] also displayed an anti-proliferative effect on cancer cells (MCF-7: IC_50_ 65.8 μM).

The extract of Sonoran Desert propolis at 22.4 ± 1.3 μM concentration displayed suitable antiproliferative activity against cancerous cell line M12.C3.F6 (murine B cell lymphoma). The antiproliferative activity through apoptosis induction was exhibited by pinobanksin and its ester derivatives, pinobanksin-3-*O*-propanoate, pinobanksin-3-*O*-butyrate, and pinobanksin-3-*O*-pentanoate [104]. Moreover, Catchpole et al. [105] evaluated the antiproliferative activity of a commercial New Zealand propolis tincture (Bio30™) reporting that the extract at 200 μg/ml concentration exerted suitable antiproliferative activity in DLD-1 cells and anti-inflammatory potential (TNF-α, COX-1, COX-2). Phenolic compounds were responsible for these activities. New Zealand wax-free Bio30™ propolis tincture solids had very high levels of the dihydroflavonoids pinocembrin and pinobanksin-3-*O*-acetate, and high levels of the dimethylallyl, benzyl and 3-methyl-3-butenyl caffeates relative to CAPE (caffeic acid phenethyl ester). Another study by Asgharpour et al. [106], described the capability of Iranian propolis to inhibit the proliferation of cancer cells. They isolated flavonoids and then, performed the *in vitr*o test using various concentrations. The isolated compounds inhibited cancer cell proliferation at 40 ± 8.9 μM and 195 ± 14.9 μM concentrations, respectively in mouth epidermoid carcinoma (KB) cell line and, at 98 μM, and 195 μM concentrations, in skin squamous cell carcinoma (A431) cell line [106]. The ethanol extract of propolis from stingless bees *Tetragonisca fiebrigi* induced cell death by necrosis at 250 and 500 μg/mL concentrations against K562 erythroleukemia cells [107]. Santos et al. [108] reported that the extract of Brazilian red propolis exerted antitumor activity at 75.15 ± 3.35, and 70.81 ± 4.18 concentrations, respectively in diverse colon cancer cell lines.

In another study, Assumpcao et al. [109] described that the *p*-coumaric acid derived from propolis displayed cytotoxic activity at 17.02, 13.94, 22.85, and 23.55 µM concentrations, respectively against triple-negative breast cancer cell lines. Besides, the ethanol extract of propolis inhibited the growth and proliferation of AGS human gastric cancer cells in a concentration range from 15 to 60 µg/ml [110]. In addition, the ethanol extract of propolis displayed significant cytotoxic activity and induced apoptosis at 50 and 100 μg/ml concentrations in human breast cancer and colon adenocarcinoma cell lines [111]. Khacha-ananda et al. [112] reported that the ethanol extract of propolis exerted cytotoxic and antiproliferative activity at a concentration of 80.96 μg/ml in HeLa cells. A study by Ishihara et al. [113] demonstrated the effects of the ethanol extract of Chinese and Brazilian propolis using different concentrations in vitro. They found that the extract at concentration > 50 μg/ml, 38 μg/ml, and 20 μg/ml induced apoptosis via growth inhibitory activity in human colon carcinoma cell lines [113]. Turan et al. [114] utilized the ethanol extract of Turkish propolis to examine the cytotoxic activity. They reported that the propolis extract at 20.7 ± 3.4 μg/ml concentration showed the highest cytotoxic activity against PC-3 cell line preventing the proliferation of cancer cells [114]. It has been reported that propolis displayed suitable cytotoxic activity at 25 and 50 μg/ml concentrations in human tongue squamous cell carcinoma cell line [115]. Besides, Lee et al. [116] described that propolis inhibited the proliferation of cells at 10 μg/ml in human rectal and colon cancer cell. Another study by Markiewicz-Żukowska et al. [117] reported that propolis reduced the growth of U87MG glioblastoma cell via nuclear factor kappa B (NF-κB) pathway at concentration ranging from 10 to100 μg/ml.

Bhargava et al. [118] stated that the isolated artepillin C from Brazilian green propolis abrogated mortalin-p53 complex, causing the activation of p53 and the growth arrest of cancer cells at 275 µM concentration in HT1080 (fibrosarcoma), A549 (lung carcinoma) and U2OS (osteosarcoma) human cell lines. In another study, Ishida et al. [119] examined the effects of isolated caffeic acid phenethyl ester from propolis on human cancer cells, SKOV3 (ovarian carcinoma), HT1080 (fibrosarcoma), A549 (lung carcinoma), HeLa (cervical carcinoma), U2OS (osteosarcoma), MCF7 and MDA-MB-231 (breast adenocarcinoma), reporting that this compound displayed suitable anticancer activity at 50 µM. A recent study by Mohamed et al. [120] reported that the ethanol extract of propolis significantly inhibited the proliferation of MCF7 cells cells at 62.24 μg/ml. Utispan et al. [121] reported that the propolis extract exerted cytotoxic activity and inhibited the proliferation of metastatic cells at 76.33 ± 1.24 µg/ml concentration in head and neck squamous cell carcinoma (HNSCC) cell lines. In a study, propolis at a concentration of 0.38 mg/mL reduced the viability of K562 erythroleukemia tumour cell line [122]. Sun et al. [123] stated that the chyrsin from propolis suppressed MDA-MB-231 breast cancer cell growth at 40 μM and 60 μM concentrations. In another study, Mutallip et al. [124] isolated pinobanksin-3-acetate from propolis, which inhibited the human colon cancer cell proliferation and induced apoptosis through up-regulation and down-regulation of multiple genes at 163.61 μg/ml concentration. Motomura et al. [125] stated that the methanol extract of propolis induced the cell cycle arrest and apoptosis in HML cells U937 at 300–500 μg/ml. It has been reported that the Brazilian red propolis produced apoptosis-like cell death and alleviated migration potential in carcinoma BCL-5637 cell line at 25, 50 and 100 μg/ml [126]. High-resolution direct-infusion mass spectrometry (HR-DIMS) was used for chemical characterization of the red propolis extract. The main components were dereplicated as follows: *m/z* 257.0764 (liquiritigenin); 269.0769 (formononetin); 271.0921 (medicarpin); 285.0718 (biochanin A); 523.1641 (retusapurpurin B). Exact mass, fragmentation pathway, and isotopic ratio were used for confirmation. Salim et al. [127] demonstrated that the ethanol extract of Egyptian propolis revealed antioxidant and antitumoral activities at 38.48 μg/ml in prostate cancer cell lines [127]. In addition, the ethanol extract of Brazilian green propolis induced apoptosis in human lung carcinoma cell line at 17.29 μg/ml [128]. Sulaiman et al. [129] experimented the effects of Iraq propolis demonstrating that its extract exhibited suitable anti-tumor activity against HL-60 and HCT-116 cell lines at 25 μg/ml.

Kamiya et al. [130] evaluated the effects of ethanol propolis extract. They observed that the extract at 10 and 20 μg/ml induced apoptosis in MCF-7 cells [130]. Frión-Herrera et al. [131] demonstrated the cytotoxic effect of ethanol propolis extract on HEp-2 human laryngeal carcinoma cell. They reported that propolis extract at 14 and 16 μg/ml displayed cytotoxic effect and induced apoptosis [131]. Motomura et al. [125] reported that the methanol propolis extract at 100, 300 and 500 μg/ml arrested the cell cycle and induced apoptosis in human leukemic U937 cells. Xuan et al. [132] stated that the ethanol extract of Chinese propolis displayed antitumoral activity against breast cancer lines MCF-7 and MDAMB-231 at 50, 100 and 200 μg/ml. Besides, the ethanol extract of Thai propolis averted the proliferation of cancer cell via apoptosis in HeLa cell line at 125 μg/mL and 250 μg/ml [133]. Czyżewska et al. [115] described that the ethanol propolis extract exerted pro-apoptotic activity against human tongue carcinoma cell line (CAL-27) at a dose of 200 μg/ml. Moreover, Vatansever et al. [134], in another study, stated that the ethanol extract of Turkish propolis induced apoptosis in MCF-7 cells at 63, 125 and 250 μg/ml via activating caspases. The ethanol extract of Brazilian propolis generated apoptosis in MCF-7 cells at 20 μg/ml [130]. It has been reported that the Brazilian propolis produced apoptosis through ROS generation in HEp-2 human laryngeal carcinoma cell at a dose of 80 μg/ml [135]. Demir et al. [100] used the ethanol extract of Turkish propolis to examine the antiproliferative and pro-apoptotic activity. They found that the propolis extract was able to alleviate mitochondrial membrane potential and enhance caspase activity in A549 cells at a concentration of 35 μg/ml [100]. Szliszka et al. [136, 137] reported that the ethanolic extract of Brazilian and Polish propolis induced apoptosis via upregulation of tumor necrosis factor-related apoptosis-inducing ligand receptor 2 (TRAIL-R2) in LNCaP prostate cancer cells at 25–50 μg/ml [136, 137]. Cardol or 5-pentadecyl resorcinol, a compound isolated from *Trigona incisa* stingless bee propolis, displayed significant antiproliferative effect, alleviated mitochondrial membrane potential, and induced apoptosis at 4.51 ± 0.76 μM concentration in SW620 human colorectal cancer cell line. Members of the Anarcadiaceae plant family are the source for this compound [138].

##### Anti-angiogeninic properties

Kunimasa et al. [139] demonstrated that the Brazilian propolis reduced angiogenesis by inducing apoptosis in human umbilical vein endothelial cells (HUVECs) at 6.25–25 μg/ml concentration. Another study by Park et al. [140] stated that the Brazilian propolis inhibited angiogenesis by preventing phosphatidylcholine-hydrolysing phospholipase C (PC-PLC) activity, p53 and ROS levels in HUVECs at 6.25–25 μg/ml concentration. The Korean propolis at 3.13–25 μg/ml concentrations suppressed angiogenesis through the inhibition of tube formation and proliferation, decreasing the number of newly formed vessels in HUVECs [141]. Moreover, Meneghelli et al. [142] reported that the ethanol propolis extract at 100–200 μg/ml concentration averted the tube-like structure formation (tubulogenesis) in HUVECs.

##### Cell migration

Red propolis induced an apoptosis-like cell death and attenuated cell migration in carcinoma BCL-5637 cell line at 25 and 50 μg/ml [126]. Xuan et al. [132], in in vitro studies, found that the green extract of propolis significantly alleviated the cells migration in breast cancer line MDA-MB-231 at 100 and 200 μg/ml. In another experimentation, Borawska et al. [143] revealed that the green propolis inhibited cells migration in glioblastoma multiforme cell line U87MG at 30 μg/ml. Finally, Taira et al. [144] evaluated the effects of ethanol propolis extract supplied by the Okinawa Yoho Bee-farm (Okinawa, Japan). They found that the propolis extract blocked the serine/threonine p21-activating kinase 1 (PAK1) and the melanogenesis by downregulating intracellular tyrosinase at 1, 6, 12 and 30 μg/ml. The main constituents were identified as prenylflavonoids by LC–MS and corresponded to nymphaeols A-C, isonymphaeol-B, and 3′- geranyl-naringenin.

#### In vivo studies

##### Chemopreventive effects

Doi et al. [145] revealed that ethanol propolis extract alleviated colon tumorigenesis in male F344 rats. According to Alizadeh et al. [146], Iranian propolis remarkably prevented the immunohistochemistry positive for β-catenin positive tumors in female Wistar rats. In a female Wistar rat model, propolis extract exhibited chemo preventive activity against rat bladder carcinogenesis [147, 148]. In a report, Salehi et al. [149] examined the Iranian propolis chemo-preventive effects. They found that the propolis extract at 100–400 mg/kg dose alleviated tongue dysplasia in male Wistar rats [149]. In another report, Xie et al. [150] stated that the ethanol propolis extract increased the N-butyl-N-(4-hydroxybutyl)-nitrosamine (BBN)-induced carcinogenesis of urinary bladder through non-mutagenicity activity in male F344 rats.

##### Immunomodulatory effects

Orsolić, Basić [151] reported that the ethanol propolis extract not only averted the tumor growth and proliferation but also enhanced the activity of tumoricidal macrophages. In another study, the ethanol propolis extract significantly suppressed the tumor growth and metastasis. In this study, the anti-metastatic activity was mediated by the immunomodulatory effects of propolis [152, 153]. Sulaiman et al. [129] reported that ethanol propolis extract at 500 or 1000 mg/kg increased the endoreduplication and p53 expression, with a reduction of mitotic cells and the Ki-67 expression in female N1-nu/nu mice. According to Missima et al*.* [154, 155], the ethanol propolis extract stimulated cytokine production and thereby, displayed stress in C57BL/6 male mice at 200 mg/kg. An investigation by Doi et al. [145] reported the the activation of NK cell cytotoxicity after the treatment with 500 mg/kg/day ethanol propolis extract in wild-type and RAG 2-deficient C57BL/6 mice.

##### Anti-angiogenic effects

Ahn et al. [156] reported that the Brazilian propolis at 2.5 and 5% exerted suppressive effects against tumor-induced angiogenesis in female ICR mice. Moreover, an experiment carried out by Chikaraishi et al. [157] used green propolis extract to determine the angiogenic effects. In this study, they found that propolis at 300 mg/kg/day significantly alleviated the retinal neovascularization in C57BL/6 mice [157]. In another study, de Moura et al. [158] showed the anti-angiogenic effects of 500 mg/kg/day water propolis extract in female Swiss mice by morphometric analysis and evaluating the hemoglobin content. They reported that the number of blood vessels decreased gradually in the treated group in comparison to the control group [158]. Dornelas et al. [159] found that the microvascular density in bladder carcinomas was lower when applying propolis extract at the concentration of 150 mg/Kg/day in female Wistar rats. Anticancer properties of propolis ethanol, water, methanol and hydroalcholic extracts and their isolated compounds are shown in Table 7.

**Table 7:** Anticancer properties of propolis ethanol, water, methanol and hydroalcholic extracts (EE, WE, ME and HAE, respectively) and their isolated compounds

Compound/extract	Model	Concentration/dose	Study type	Mechanism	Origin	References
Hyperibone A	HeLa tumor cells	IC_50_ = 0.18 µM	In vitro	Cytotoxic	Brazil	[95]
Nemorosone	HeLa tumor cells	IC_50_ = 3.3 µM	In vitro		Cuba	[95]
Luteolin	Human colon cancer (HCT-116)	IC_50_ = 115.68 μM	In vitro	Cytotoxic/apoptosis	Serbia	[96]
Luteolin	Human triple negative breast cancer (MDA-MB-231) cell lines	IC_50_ = 66.86 μM	In vitro			
Myricetin	Human triple negative breast cancer (MDA-MB-231) cell lines	IC_50_ = 114.75 μM	In vitro	Inhibition of cell growth and apoptosis		
Galangin	Human colon cancer and human triple negative breast cancer cell lines	IC_50_ = 50 μM	In vitro	Apoptosis		
EE	glioblastoma cells cancer line	IC50 = 92.2 µg/mL	In vitro	Cytotoxic	Mexico	[98]
(7′′*R*)-8-[1-(4′-Hydroxy-3′-methoxyphenyl) prop-2-en-1-yl] galangin	PANC-1 humanpancreatic cell line	IC_50_ = 4.6 μM	In vitro	Apoptosis-like morphological changes/Cytotoxic		[97]
Chrysin, pinobanksin, pinobanksin-3-*O*-propanoate, pinobanksin-3-*O*-butyrate, pinobanksin-3-*O*-pentanoate, pinobanksin-3-*O*-hexanoate	B-cell lymphoma cell line	IC_50_ = 49.1 µM; 52.1 µM; 67.0 µM; 49.9 µM; 51.3 µM; 76.6 µM	In vitro	Antiproliferative activity through apoptotic induction	Sonaran	[99]
Turkish propolis EE	Human adenocarcinomic alveolar basal epithelial (A549) cancer	IC_50_ = 31.7 µg/mL	In vitro	Cell cycle arrest (G_1_ phase), induction of endoplasmic reticulum stress, caspase activity, and apoptosis; decrease ofmitochondrial membrane potential	Turky	[100]
Turkish EE	human normal foreskin fibroblast cells	IC_50_ = 76.9 µg/mL	In vitro	Cytotoxic activity		
Turkish EE	Human adenocarcinomic alveolar basal epithelial (A549) cancer	31.7 and 57.1 µg/mL	In vitro	Increase of caspase activity; decrease of mitochondrial membrane potential; up-regulation CHOP mRNA expression; cell cycle arrest (G_1_phase)—apoptosis	Turky	[100]
EE	HCT 116 Colon Cancer and Me45 Malignant Melanoma Cells	IC_50_ = 100 µM	In vitro	Anti-proliferative and pro-apoptotic Effect	Poland	
Propolone A-B	Ovarian cancer cell line	LC_50_ = 19.1 µM and 29.9 µM	In vitro	Anti-proliferative	Brazil	[102]
Novel 2-phenoxychromone; 3-*O*-methylquercetin; 3,6,4′-trimethoxychrysin; 3,6-dimethoxyapigenin	DLD-1 (human colon cancer),	IC_50_ = 65.8; 16.2; 17.1; 31.0 μM	In vitro	Anti-proliferative effect; cell growth inhibition	Brazil	[103]
	MCF-7 (human breast cancer) and	IC_50_ = 174.4; 16.7; 50.5; 41.9 μM				
	A549 (human lung cancer) cancer cell lines	IC_50_ = 81.9; 34.2; 19.9; 47.0 μM				
Sonoran Desert EE	Cancerous cell line M12.C3.F6 (murine B cell lymphoma)	IC_50_ = 22.4 μM	In vitro	Antiproliferative	Sonoran Desert	[104]
Caffeic acid and pinocembrin	Human colorectal adenocarcinoma DLD-1 cells	IC_50_ = 200 μM	In vitro	Antiproliferative	New Zealand	[105]
Ardabil EE and quercetin	Mouth epidermoid carcinoma (KB) cell line	IC_50_ = 40 mM and 195 μM	In vitro	Cell proliferation inhibition	Iran	[106]
Ardabil EE and quercetin	Skin squamous cellcarcinoma (A431) cell line	IC_50_ = 98 μM, and 195 μM	In vitro	Cell proliferation inhibition	Iran	[106]
EE from stingless bees *Tetragoniscafiebrigi*	K562 erythroleukemia cells	IC_50_ = 250 and 500 μg/mL	In vitro	Necrosis	Brazil	[107]
Brazilian red EE	Colon cancer cell lines (human colorectal adenocarcinoma and human colorectal carcinoma)	IC_50_ = 75.15 and 70.81 μg/mL	In vitro	Antitumor activity	Brazil	[108]
*p*-Coumaric acid	Four triple-negative breast cancer cell lines (BT-20, BT-549, MDA-MB-231, and MDA-MB-436 cells)	IC_50_ = 17.02, 13.94, 22.85, and 23.55 µM	In vitro	Cytotoxic; decrease of cell viability	Brazil	[109]
( −)-Epigallocatechin-3-gallate	BT-20, BT-549, MDA-MB-231, and MDA-MB-436 cells,	IC_50_ = 20.10, 19.16, 24.97 and 18.16 µM	In vitro	Cytotoxic; decrease of cell viability	Brazil	[109]
EE	AGS human gastric cancer cell	60, 30, and 15 µg/mL	In vitro	Cell growth and proliferation inhibition	Iran	[110]
	Human breast cancer, colon adenocarcinoma, epithelial colorectal adenocarcinoma, murine melanoma	50 and 100 μg/mL	In vitro	Cytotoxic/apoptosis	India	[111]
	HeLa cervical cancer cells	IC_50_ = 80.96 μg/mL	In vitro	Cytotoxic/anti-proliferative effect	Thailand	[112]
EE	Human colon carcinoma cell lines CaCo2	IC_50_ = 50 μg/ml	In vitro	Growth inhibitory activity by apoptosis	China	[113]
	Human colon carcinoma cell lines CaCo2	20 μg/ml	In vitro	Decrease of cells in G1, S and G2-M phases, modulation of p53 protein	China	[113]
	HCT116	38.9 μg/ml	In vitro	Growth inhibitory activity by apoptosis	Brazil	[113]
EE	Human colon carcinoma cell lines CaCo2	> 50	In vitro	Growth inhibitory activity by apoptosis	Brazil	[113]
Turkish EE	PC-3 cell line	IC_50_ = 20.7 μg/mL	In vitro	Cytotoxic activity		[114]
Flavonoids and phenolic acid	Human tongue squamous cell carcinoma cell line	25 and 50 μg/mL	In vitro	Activation of caspases-3, -8 and -9		[115]
	Human rectal and colon cancer cell	10 μg/mL	In vitro	Proliferation inhibition		[116]
	Human U87MG glioblastoma cell	TMZ (10–100 μM), EE (10-100 μg/mL)	In vitro	Glioblastoma cell growth inhibition NF-κB activity down-regulation		[117]
Artepillin C	HT1080 (fibrosarcoma), A549 (lung carcinoma) and U2OS (osteosarcoma) human cell lines	IC_50_ = 275 µM	In vitro	Abrogation of mortalin-p53 complexes causing the activation of p53	Brazil	[118]
Caffeic acid phenethyl ester	Human cancer cells, SKOV3 (ovarian carcinoma), HT1080 (fibrosarcoma), A549 (lung carcinoma), HeLa (cervical carcinoma), U2OS (osteosarcoma), MCF7 and MDA-MB-231 (breast adenocarcinoma	50 µM	In vitro	Down-regulation of mortalin and up-regulation of GADD45α and p53 tumor suppressor proteins	New Zealand	[119]
EE	MCF7 cells	IC_50_ = 62.24 μg/mL	In vitro	Proliferation inhibition		[120]
DMEP-A-C	head and neck squamous cell carcinoma (HNSCC) cell lines	IC_50_ = 76.33 µg/mL	In vitro	Cytotoxic activity; metastatic proliferation inhibition		[121]
*P*. *droryana* propolis	the K562 erythroleukemia tumour line	IC_50_ = 0.38 mg/mL	In vitro	Cell viability decrease		[122]
Chyrsin	MDA-MB-231breast cancer cell	40 μM and 60 μM	In vitro	Up-regulation of p21(waf1/cip1) gene expression and inhibition of histone deacetylase 8	China	[123]
	xenograft animalmodel	90 mg/Kg/day *per os*		Cell growth suppression		
Pinobanksin-3-acetate	human colon cancer	IC_50_ = 163.61 μg/mL	In vitro	proliferation inhibition and apoptosis induction through up-regulation and down-regulation of multiple genes involved in cell apoptosis, cytokinetics, colorectal carcinogenesis, Wnt, and calcium signaling		[124]
ME	HML cells U937	300–500 μg/mL	In vitro	Dose-dependent decrease of Bcl-2 expression, no changes in Bax expression, apoptosis		[125]
Liquiritigenin, formononetin, medicarpin, biochanin A, retusapurpurin	Carcinoma BCL- 5637	25, 50 and 100 μg/mL	In vitro	Increase of Bax/Bcl-2 ratio levels	Brazil	[126]
EE	Prostate Cancer cell lines PC-3	IC_50_ = 38.48 μg/mL	In vitro	Slight increase on Bax mRNA	Egypt	[127]
EE	Human lung carcinoma cell line	1/4 IC_50=_ 17.29 μg/mL	In vitro	Decrease of mitochondrial membrane potential by overexpression of pro-apoptotic genes (Bax and Noxa) and decrease of the Anti-apoptotic gene Bcl-XL	Brazil	[128]
WE^°^	HL-60 and HCT-116 cell lines	25 μg/mL	In vitro	Increase of Bax	Iraq	[129]
EE	Breast Cancer lines MCF-7	10 and 20 μg/mL	In vitro	Increase of Bax mRNA and decrease ofBcl-2 mRNA	Brazil	[130]
EE	HEp-2 human laryngeal carcinoma cell	1/4 IC_50_ = 14 and 16 μg/mL	In vitro	Down-regulation of Bcl-2 and Bcl-XL mRNA; Up-reguation of Bax; apoptosis	Brazil	[131]
ME	HML cells U937	100, 300 and 500 μg/mL	In vitro	Caspase-3 activation	Japan	[125]
EE	Breast Cancer lines MCF-7 and MDAMB- 231	50, 100 and 200 μg/mL	In vitro	Caspase-3 activation	Chinese	[132]
EE	HeLa	Nan: 125 μg/mL Chiang Mai: 250 μg/mL	In vitro	Caspase-3 activation	Thailand	[133]
EE	Human tongue carcinoma cell line (CAL-27)	200 μg/mL	In vitro	Caspase − 3,-8 and -9 activation	Poland	[115]
EE	Breast Cancer lines MCF-7	63, 125 and 250 μg/mL	In vitro	Activation of caspase-6 than caspases-8 and 9	Turkey	[134]
EE	Breast Cancer lines MCF-7	20 μg/mL	In vitro	Caspase-3 activation	China and Brazil	[130]
	HEp-2 human laryngeal carcinoma cell	IC_50_ = 80 μg/mL	In vitro	Apoptosis due to ROS generation and caspase-3 activation	Brazil	[135]
EE	Human lung carcinoma cell line A549	35 μg/mL	In vitro	Mitochondrial membrane potential decrease and caspase activity increase	Turkey	[100]
EE	LNCaP prostate cancer cells	25–50 μg/mL	In vitro	Up-regulation of TRAIL-R2		[136, 137]
Cardol	SW620 human colorectal cancer cell line	IC_50_ = 4.51 ± 0.76 μM	In vitro	Increase of caspase-3 and -9 activityand PARP; apoptosis; mitochondrial membrane potential decrease; antiproliferative effect; G_0_/G_1_ cell cycle arrest	Indonesian propolis	[138]
EE	HUVECs	6.25–25 μg/mL	In vitro	Apoptosis in tube-forming endothelial cells through inactivation of survival signal ERK1/2	Chinase	[139]
EE	HUVECs	6.25–25 μg/mL	In vitro	Decrease of PC-PLC activity, p53 and ROS levels	Brazil	[140]
EE	HUVECs	3.13–25 μg/mL	In vitro	Angiogenesis suppression through inhibition of tube formation and proliferation; decrease of the number of newly formed vessels	Korean	[141]
HAE	HUVECs	100–200 μg/mL; 50–450 mg/Kg	In vitro	Inhibition of the tube-like structure formation (tubulogenesis)		[142]
EE	Carcinoma BCL-5637	25 and 50 μg/mL	In vitro	Cell migrationinhibition	Brazil	[126]
EE	Breast Cancer line MDA-MB -231	25, 50, 100 and 200 μg/mL	In vitro	Cells migration inhibition	China	[132]
EE	Glioblastoma multiforme cell line U87MG	30 μg/mL	In vitro	Cell migration inhibition	Poland	[143]
EE	Human lung cancer A549 cell, melanoma cell line B16F10	1, 6, 12 and 30 μg/mL		Block of PAK1 and melanogenesis by down-regulation of intracellular tyrosinase activity	Japan	[144]
EE	Colon Tumorigenesis in male F344 rats	1% EE or WE, basal diet, 25 weeks	In vivo	Tumorigenesis decrease	Brazil	[145]
EE	Gastric cancer in Male Wistar rats	Enriched feed, 36 weeks	In vivo	Significantly decrease of IHC β-catenin positive tumors	Iran	[146]
EE	Bladder cancer in female Wistar rats	150 mg/Kg/day, intragastric, 40 weeks	In vivo	Chemo-preventive effects	Brazil	[147, 148]
EE	Dysplasia of tongue in male Wistar rats	100–400 mg/Kg, intraperitoneall, 20 weeks	In vivo	Tumor decrese	Iran	[149]
EE	Bladder Carcinogenesis in male F344 rats	0.125 to 1%, dietary administration, 32 weeks	In vivo	Enhances of BBN-initiated urinary bladder carcinogenesis via non-mutagenic mechanisms	Brazil	[150]
EE	Male Swiss albino mice	50 mg/Kg, gastric incubation, 7 days	In vivo	Tumor growth and proliferation inhibition; Increase of macrophages tumoricidal activity	Brazil; Zagreb, Croatia	[151]
EE	Male and female CBA inbred mice	50 or 150 mg/Kg, gauge administration, 3 days	In vivo	Suppression of tumor growth and metastases; Antimetastatic activity mediated by immunomodulatory effects	Brazil, Zagreb, Croatia	[152, 153]
EE	Female athymic Fox N1-nu/nu mice	500 or 1000 mg/Kg/day, p.o., 3 weeks	In vivo	Mitotic cell and Ki-67 expression decrease and increase in endoreduplications and p53 expression	Mosul, Iraq	[129]
EE	C57BL/6 male mice	200 mg/Kg, 14 days	In vivo	Transcription of stress stimulated Th1 cytokine (IL-2 and IFN-γ) and Th2 cytokine IL-10	Brazil	[154, 155]
EE	Female ICR mice	2.5 and 5%, oral administration, 6 days	In vivo	Suppressive effects on tumor-induced angiogenesis	Brazil	[156]
EE	OIRM in C57BL/6 mice	300 mg/Kg/day, subcutaneous administration, 5 days	In vivo	Suppression of retinal neovascularization	Brazil	[157]
EE	Female Swiss mice	500 mg/Kg/day, orally, 14 days	In vivo	Progressively increase of blood vessel number	Brazil	[158]
EE	Bladder cancer in female Wistar rats	150 mg/Kg/day, 40 weeks	In vivo	Decrease of microvascular density	Brazil	[159]

### Analgesic effects

One of the most prevalent complaints seen in medical practice is pain [160]. Following tissue damage and/or injury, nociceptors (specialist peripheral sensory neurons) are triggered by noxious stimuli (chemical, mechanical, and thermal stimuli). Muscle, cutaneous tissues, connective tissues, arteries, viscera, and bone are all places where nociceptors may be found [161]. The release of inflammatory mediators, which can lower the threshold of nociception, and direct activation of nociceptive afferent fibers owing to pH lowering may both be involved in the acetic acid-induced nociceptive response [162]. Propolis red extract showed analgesic effect on mice model [163] via suppression of NF-kB [164]. Bulgarian propolis was reported to inhibit the contraction of trachea smooth muscle induced by histamine, capsaicin, and carbachol in vitro displaying analgesic effect [165]. Furthermore, Brazilian propolis exhibited anti-nociceptic effect and reduced paw oedema via activating the opioid receptors in rats [166]. Arachidonic acid ethyl ester, a bioactive substance isolated from Cameroonian propolis, reduced acute central pain sensation [167].

Propolis and its chemical constituents presented antinociceptive action on inflammatory and neurogenic pain without motor side effects [168]. Also, one the main chemical analogues of propolis, formononetin, showed antinociceptic effect [168]. Chinese propolis also showed central and peripheral antinociceptive properties due to the presence of flavonoids [169]. In the writhing model, propolis reduced the number of writhes, leading to antinociceptive effect [170].

Propolis demonstrated antinociceptic properties via two possible ways against acetic acid writhing model, i. by local peritoneal receptors; ii. by inhibition of prostaglandin synthesis or action [171, 172]. Furthremore, propolis was used to treat a wide variety of gynecologic, dental, and dermatological issues, where pain is a most frequent issue [173]. The release or production of mediators (e.g., serotonin, kinins, prostaglandin and aminoacids) from neurons or injured tissues is involved in the inflammatory process and in pain processing responses [174, 175].

A number of phytochemical constituents were identified in differents propolis samples such as chalcones and dihydrochalcones [176, 177], flavanones, flavones and flavonols, hydrocarbons esters ethers, terpenoids, lignans, steroids [49, 178]. Brazilian red and, green propolis, Chinese propolis, Bulgarian propolis, Iranian propolis and Cameroonian propolis have the ability to reduce pain sensation in mice/rat models [165, 167, 169, 170]. Brazilian propolis suppressed acetic acid-induced pain and increased the pain threshold reducing the nociceptive response [160]. As a result, the high flavonoids content in green Brazilian propolis could explain these anti-inflammatory properties [179]. Propolis extract promotes the down-regulation of pro-inflammatory cytokines (e.g., interleukin (IL)-1, TNF-α, and IL-6), inhibits the purine nucleoside phosphorylase, and increase the anti-inflammatory IL-10 according to previous results [180, 181]. Several investigations have tried to understand the processes behind the analgesic action of propolis [182]. Previous studies have established that propolis contains several polyphenols, terpenoids and lignans such as artepillin C, neovestitol, vestitol [165], pinocembrin, chrysin, galangin, kaempferol, quercetin, isoliquiritigenin, acetyl-1-coumaroyl-3-cinnamoylglycerol, (+)-2-acetyl-1-feruloyl-3-cinnamoylglycerol, ( −)-2-acetyl-1-feruloyl-3-cinnamoylglycerol, 2-acetyl-1,3-dicinnamoylglycerol and ( −)-2-acetyl-1-(*E*)-feruloyl-3-(3″,16″)-dihydroxy-palmitoylglycerol, 3,3-dimethylallyl caffeate, caffeic acid phenethyl ester, which showed anti-inflammatory properties in different experimental systems [183–186]. It has been reported that flavonoids suppressed the release of arachidonic acid and interact with GABA-ergic, serotonin and L-arginine–nitric oxide (NO) system [187].

### Anti-inflammatory activity

The anti-inflammatoy activities of propolis are shown in Table 8. Propolis has great impact on inflammation [188], because it up-regulates or down regulates several inflammatory mediators [189]. A number of compounds were isolated from propolis, which have anti-inflammatory effects [190]. Artepillin C showed anti-inflammatory potential in adipocytes via down-regulation of adiponectin mediated by TNF-α and c-Jun N-terminal kinase (JNK) pathways [189], and decreasing inflammatory cells through modulation of matrix remodeling [190]. Furthermore, in an in vivo experimental model, artepillin C, reduced oedema by decreasing neutrophils, NO and prostaglandin E2 (PGE_2_) release [191] and by down-regulation of the NF-κB pathway, which help to attenuate the release of pro-inflammatory cytokines such as TNF-α [191]. These data were confirmed by Szliszka et al. [179], who demonstrated that artepillin C have the ability to in vitro down-regulate the inflammatory chemokines IL-1β, IL-3, IL-4, IL-5, IL-9, IL-12 p40, IL-13, IL-17, TNF-α, G-CSF, GMCSF, MCP-1, MIP-1α, MIP-1β, RANTES by modulating the NF-κB pathway [179]. Other studies reported that propolis extract and artepillin C decreased cysteinyl leukotrienes and histamine in peripheral leukocytes [192, 193]. Moreover, propolis extract inhibited granuloma and exudate formation [194]. Ethanol propolis extract with CAPE exerted anti-inflammatory effects in wistar rat model through inhibition of carrageenin oedema, pleurisy, and adjuvant arthritis [195]. Further studies showed that propolis extract accelerated the down-regulation process of pro-inflammatory cytokines release (e.g., IL-1β, TNF-α and IL-6), inhibited purine nucleoside phosphorylase and increased the anti-inflammatory IL-10 in macrophage cell line [180, 181]. Several inflammatory illnesses have been linked to excessive production of these mediators [196].

**Table 8:** Anti-inflammatory properties of propolis ethanol and water extract (EE and WE, respectively) and their isolated bioactive compounds

Compound/extract	Origin	Concentration /Dose	Study type	Test system	Mechanism	References
Artepillin C	Brazil	25 μM	In vitro	3 T3-L1 adipocytes	Inhibition of TNF-α Down regulation of adiponectin, increase of PPAR-γ activity; inhibition of TNF-α-induced JNK signaling	[189]
	Brazil	500 mg/Kg, orally, 14 days	In vivo	Female Swiss mice	Decrease of inflammatory Cells, modulation of matrix remodeling	[190]
	Brazil	10 mg/Kg, single oral dose	In vivo	Male Swiss mice	Decrease of oedema, reduction of neutrophils number, decrease of prostaglandin E2 level	[191]
	Brazil	10 μM 100 μM	In vitro	RAW 264.7 cells	Reduction in NO synthesis	[191]
				HEK 293 cells	Decrease of the NF-κB levels	
		50 μM 100 μM	In vitro	RAW 264.7 cells	Inhibition of IL-1β, IL-3, IL-4, IL-5, IL-9, IL-12 p40, IL-13, IL-17, TNF-α, G-CSF, GMCSF, MCP-1, MIP-1α, MIP-1β, RANTES, KC and NF-κB pathway	[179]
	Brazil	30 μg/mL 100 μg/mL	In vitro	Peripheral leukocytes of human blood	Decrease cysteinyl leukotrienes and histamine	[192]
Propolis extract	-	10 mg/kg	In vivo	Carrageenan rat paw oedema rat model	Reduce prostaglandins, leukotrienes and histamine	[193]
		50 and 100 mg/Kg, orally, 7 days	In vivo	Carrageenin paw edema rat model	Inhibitory effect on granuloma and exudate formation	[194]
EPE with CAPE		100–600 mg/Kg, 35 days	In vivo	Male Wistar rat model	Inhibit carrageenin oedema, carrageenin pleurisy and adjuvant arthritis	[195]
Propolis extracts	Cameroon	30 μg/mL	In vitro	THP-1-derived macrophage cells	Inhibits pro-inflammatory IL-1β, TNF-α and IL-6, increase IL-10 and inhibition of purine nucleoside phosphorylase	[181]
Neovestitol and vestitol	Brazil	10 mg/Kg	In vivo	Male Balb/c mice	Neutrophil migration	[197]
Bulgarian Propolis	Bulgary	ID50 = 2.5 0.4 mgkg-1		Swiss male mice		[165]
Apigenin		50 mg/Kg, intraperitoneally, 30 days	In vivo	LPS- induced Male mice	Inhibit inflammatory cytokines, ERK1/2, NF-kB activation, and neutrophill migration	[204, 205]
Galangin		50 μM	In vitro	RAW 264.7 murine macrophages	Inhibit inflammatory cytokines IL-1β and IL-6, and proinflammatory genes, such as iNOS, ERK1/2, NFkB p65 activation, and neutrophill migration	[203, 206]
pinocembrin		20 or 50 mg/Kg *ip*	In vitro	RAW macrophage cells	Modulate the production of TNF-α, IL-1β, IL-6 and IL-10 via inhibiting the phosphorylation of IκBα, ERK1/2, JNK and p38MAPK	[212]
Formononetin		10 mg/Kg, orally	In vivo	Carrageenan-induced hindpaw oedema rat model	Inhibition of leukocyte migration and activation of NF-κB	[168]
HERP		10 and 30 mg/Kg, orally				
Formononetin		10 mg/Kg	In vitro	INS-1 rat insulinoma cell line	Prevent IL-1β and cytokine-induced apoptotic signaling, reducethe Bax/Bcl-2 ratio and caspase-3 activity; activateNF-κB, decease NO release	[210]
Neovestitol and vestitol		10 mg/Kg	In vivo	LPS mice model	Inhibition of neutrophil migration, rolling and adhesion; inhibition of CXCL1/KC and CXCL2/MIP2 levels and neutrophil chemotaxis via blocking calcium influx	[198, 199]
Isoliquiritigenin		10 mg/mL	In vitro	Human primary endothelial cells	Inhibit adhesion of neutrophil, ICAM-1, VCAM1,E-selectin and TNF-α expression, translocation of the p65 subunit of NF-κB by blocking the phosphorylation and subsequent degradation of IκBα	[213]
Daidzein		2, 4, 8 mg/Kg, i.p	In vivo	Adult male Sprague–Dawley rats model	Inhibited macrophages and neutrophils infiltration, attenuated MPO activity, inhibited TLR4,MyD88 protein and NF-κB activation	[214]
Chinese propolis extract	China	15 μg/mL	In vitro	RAW 264.7 cells	Inhibit the production of NO, IL-1*β*, and IL-6; suppress the phosphorylation of I*κ*B*α* and AP-1; block the activation of NF-*κ*B in TNF-*α*-stimulated HEK	[8]
Senegalese propolis	Senegal	30 μg/mL	In vitro	J774.1 cells	Inhibited iNOS	[216]
EE	China	25 and 100 mg/Kg, orally, 3 days	In vivo	RAW 264.7 cells/endotoxemic mice	Suppress the secretion of LPS-stimulated inflammatory cytokines, such as IL-6, IL-10, MCP-1, TNF-α and IL-12p70 production	[447]
Propolis, coumaric and cinnamic acids		50 and 100 mg/well	In vivo*/ vitro*	Male BALB/c mice/ Peritoneal macrophages	Inhibited IL-10 and IL-6	[180]
Propolis extract		10 μg/mL	In vitro	RAW 264.7 mouse macrophage cells	Inhibition of IL-1β, IL-6 and COX-2 mRNA expression	[211]
2-Acetyl-1-coumaroyl-3-cinnamoylglycerol, ( +)-2-acetyl-1-feruloyl-3-cinnamoylglycerol, ( −)-2-acetyl-1-feruloyl-3-cinnamoylglycerol, 2-acetyl-1,3-dicinnamoylglycerol and ( −)-2-acetyl-1-(*E*)-feruloyl-3-(3″(ζ),16″)-dihydroxy-palmitoylglycerol		10 and 100 μM	In vitro	Lipopolysaccharide (LPS)-stimulated RAW 264.7 mouse macrophage cells	Inhibitory effects on interleukin IL-1β, IL-6, and COX-2	[215]
3,3-Dimethylallyl caffeate in mixture with isopent-3-enyl caffeate		IC_50_ = 0.49 μM	In vivo	Ear edema test in mice	Anti-inflammatory activity	[218]
Arachic acid ethyl ester		50.0 mg/Kg	In vivo	Carrageenan-induced paw edema, xylene-induced ear edema mice	Anti-inflammatory effect	[167]
Polyphenol-rich propolis extracts		100 mg of CPPE/Kg	In vitro	Lipopolysaccharide (LPS)-challenged mice/HEK 293 T cells	Suppressed NF-κB activation via inhibiting the ubiquitination of TRAF6; inhibitory effects on IκBα phosphorylation and p65 nuclear translocation; lowered serum IL-6, MCP-1, IFN-γ, TNF-α and IL-12p70	[208]
2-Hydroxy-8-prenylbiochanin A		IC_50_ = 23.3 µg/mL	In vitro	Lipopolysaccharide (LPS) stimulated J774.2 mouse macrophages	NO inhibition	[219]
β-Amyrine acetate		IC_50_ = 4.3 µg/mL			ROS inhibition	
Lupeol acetate		IC_50_ = 1.1 µg/mL				
South Brazilian organic propolis	Brazil	0.1, 1, and 10 μg/mL	In vitro	RAW 264.7 macrophages	Decreased NF-kB activation and TNF-α release	[207]
3′,4′-Dihydroxy-4-methoxydalbergione, 4-methoxydalbergion, cearoin, and chrysin	Nepal	30 μg/mL	In vitro	Bone marrow-derived mast cells (BMMC) and cell cultures	Inhibited IL-33-induced mRNA expression of inflammatory genes including IL-6, TNFα and IL-13 and activation of NF-κB	[182]
WE		50 mg/kg, i.p	In vivo	Male Wistar albino rats	Reduced ciliary body NF-κB/p65 immunoreactivity and AH levels of HIF-1α and TNF-α	[201]
EE		50 μL	In vitro	Hyaluronidase assay	Inhibited the hyaluronidase enzyme	[107]
Brazilian organic propolis	Brazil	10 μg/mL	In vivo	C57BL/J6 SPF male mice	Reduced NF-κB activation, TNF-α release, and neutrophil migration	[448]
EE		1 mg/Kg	In vivo	Carrageenan-induced paw oedema in rats model	Decreased PGE_2_ and NO levels, inhibited the IL-6 increase	[217]
Daidzein, formononetin Aand biochanin A		4.68 μg/mL, 31.81 μg/mL, 9.58 μg/mL	In vivo	Adult male Wistar rats	Anti-inflammatory	[209]
Luteolin, galangin, quercetin		0.5 mg	In vivo	Croton oil-induced oedema in rats	Inhibited IL-1β and TNF-α	[202]
Lebanese propolis EE	Lebanon	30 μg/mL	In vitro	LPS-activated RAW monocytes	Inhibited COX-2 and iNOS protein expression, as well as PGE_2_ and NO release	[222]
Malaysian propolis	Malaysia	300 mg/kg b.w	In vivo	Male Sprague Dawley rats	Reduced malondialdehyde, NFkB (p65), TNF-α, IL-1β	[221]
CAPE		30 mg/kg	In vivo	High-fat diet-induced obesity in mice	Reduced the induction of the inflammatory pathway, c-jun-N-terminal kinase, NF-κB, COX-2 expression	[186]
		100 and 200 mM	In vitro	Human middle ear epithelial cells	Inhibited upregulation of TNF-α and COX-2	[185]
		2.8 × 10^–4^–2.8 µgmL–	In vitro	Murine monocyte/macrophage J774 cell line	Inhibited COX-1 and COX-2	[184]
		30 mg/kg	In vivo	Gy gamma whole-body irradiation of rats	Decreased IL‐6R, IL‐6, IL‐8, enhanced IL10, suppressed cytokine signaling-3,	[223]
Caffeic acid		10 μg/mL	In vitro	Raw 264.7 macrophages	Inhibited NO production, p38 MAPK, JNK1/2 and NF-κB	[224]
EEP, chrysin, galangin, kaempferol quercetin		30 μg/mL	In vitro	J774A.1 macrophages	Suppressed both IL-1β mRNA (*P* < 0.02) and iNOS mRNA (*P* < 0.001) expression, decreased the IL-1β mRNA level and IL-1β protein concentration	[183]
Neovestitol		0.22 µM	In vitro	RAW264.7 murine macrophages	Inhibited NO production and reduced GM-CSF, IFN-γ, IL-1β, IL-4, TNF-α and IL-6 levels, increased IL-10 production, down-regulated genes related to nitric oxide production, NF-κB, IL-1β, and TNF-α signaling pathways	[200]
Okinawa propolis	Japan	200 μM	In vitro	RAW 264.7 cells	Reduced iNOS, COX-2, and PGE_2_	[220]

Vestitol, neovestitol, and Bulgerian propolis extract suppressed the neutrophil migration [165, 197]. Besides, in a lipopolysaccharide (LPS)-induced inflammatory mouse model, vestitol and neovestitol blocked neutrophil migration, rolling, and adhesion, as well as inhibiting the levels of the chemokines CXCL1/KC and CXCL2/MIP2 and neutrophil chemotaxis by preventing calcium influx [198, 199]. Neovestitol inhibited NO production and reduced GM-CSF, IFN-γ, IL-1β, IL-4, TNF-α and IL-6 levels, increased IL-10 production and down-regulated genes related to NO production, NF-κB, IL-1β, and TNF-α signaling pathways [200].

Brazil propolis extract inhibited inducible nitric oxide synthase (iNOS) gene expression [164], TNF-α release, and neutrophil migration via reducing NF-κB activation [201]. Apigenin, luteolin, quercetin and galangin, active constituents isolated from propolis, down-regulated inflammatory cytokines such as IL-1β and IL-6, TNF-α and inflammatory genes, such as iNOS, extracellular signal-regulated kinase 1/2 (ERK1/2) and NF-kB and inhibited neutrophill migration [202–206]. Formononetin also suppressed the activation of NF-kB pathway via ubiquitination of TRAF6 [168, 207, 208] and arrest IL-1β, cytokine-induced apoptotic signaling by blocking Bax/Bcl-2 ratio, caspase-3 activity and NO release, exerting anti-inflammatory effect [209, 210]. Furthermore, both IL-1 and iNOS mRNA expression were decreased by chrysin, galangin, kaempferol, and quercetin in macrophages [183].

Pinocembrin inhibited the phosphorylation of IkBα, ERK1/2, JNK, and p38/MAPK pathways, which controlled the production of inflammatory cytokines (TNF-α, IL-1, IL-6, and IL-10) [180, 211, 212]. Isoliquiritigenin inhibited neutrophil adhesion, adhesion molecule 1 (ICAM-1), vascular cell adhesion molecule 1 (VCAM-1) and E-selectin expression and translocation of the p65 subunit of NF-κB by blocking the phosphorylation and subsequent degradation of IκBα in human primary endothelial cells [213]. Daidzein also blocked the activation of NF-κB, inhibiting macrophages and neutrophils infiltration in the inflammatory response. Furthermore, it attenuated myeloperoxidase (MPO) activity and MyD88 protein [209, 214].

Propolis extract from China also exerted anti-inflammatory properties via attenuating the production of NO, IL-1*β*, and IL-6 mediated by the phosphorylation of I*κ*B*α*, AP-1 and NF-*κ*B pathways [8, 215]. Senegalese propolis blocked the inflammatory gene iNOS [216]. Propolis extract and the isolated bioactives 2-acetyl-1-coumaroyl-3-cinnamoylglycerol, ( +)-2-acetyl-1-feruloyl-3-cinnamoylglycerol, ( −)-2-acetyl-1-feruloyl-3-cinnamoylglycerol, 2-acetyl-1,3-dicinnamoylglycerol and ( −)-2-acetyl-1-(*E*)-feruloyl-3-(3″,16″)-dihydroxy-palmitoylglycerol down-regulated the IL-1β, IL-6, cyclooxygenase (COX)-2 mRNA expression in mouse macrophage cells [211, 215]. Propolis aqueous extract decreased ciliary body NF-kB/p65 immunoreactivity as well as hypoxia inducible factor (HIF-1) and TNF-α levels in the aqueous humor [201]. Ethanol extract of propolis attenuated hyaluronidase enzyme [107]. Hu et al. [217] demonstrated that ethanol extract of propolis decreased PGE_2_ and NO levels and inhibited IL-6 release in carrageenan-induced paw oedema in a rat model. Furthermore, a mixture of three bioactive compound isolated from Mexican propolis (3,3-dimethylallyl caffeate, isopent-3-enyl caffeate and arachic acid ethyl ester) reduced inflammation in ear and paw edema [167, 218]. Moreover, 2-hydroxy-8-prenylbiochanin A, β-amyrine acetate and lupeol acetate increased the NO inhibition in macrophages [219]. In bone marrow-derived mast cells (BMMC), 3′,4′-dihydroxy-4-methoxydalbergione, 4-methoxydalbergion, cearoin, and chrysin isolated from Nepalese propolis, reduced the IL-33-induced activation of NF-kB [182]. Ethanol extract of Lebanese and Malaysian propolis induced anti-inflammatory reaction through blocking TNF-α, IL-1β, COX-2 PGE2, NO release and iNOS protein expression, as well as malondialdehyde (MDA) and NF-kB (p65) expression [220–222]. Furthermore, CAPE suppressed the activation of NF-kB p65, decreasing the inflammatory pathway. In addition, it reduced the expression of JNK, COX-1 and COX-2, and TNF-α in obese mice [184–186] and the release of IL‐6 and IL-8, enhanced the anti-inflammatory cytokines IL-10 and suppressed the cytokine signaling-3 pathway [223]. Caffeic acid prevented macrophages from producing NO, via activating p38 MAPK, JNK1/2, and NF-kB [224].

### Antidiabetic effects

Diabetes is a prevalent chronic metabolic health condition [225]. α-Amylase and α-glucosidase are enzymes necessary for the uptake of glucose from starch or maltose. α-glucosidase is essential for disaccharides hydrolisis and, suppressing this enzyme, it is possible to attenuate the blood glucose absoption [226]. Many drugs, that inhibit α-glucosidase have been shown to reduce post-prandial hyperglycemic peaks, making them effective in the treatment of type 2 diabetes. However, a variety of side effects such as stomach pains or diarrhea may make it difficult for patients to stick to their treatment regimen [226]. As a result, natural compounds that inhibit α-glucosidase might be promising therapeutic candidates for the treatment of diabetic mellitus. In this sense, propolis showed hypoglycemic effect by blocking α-glucosidase (IC_50_ 3.77 µM) [220, 227]. Many compounds isolated from propolis such as astrapterocarpan, medicarpin and 8-prenylnaringenin exert antidiabetic effect via suppressing α-amylase and α-glucosidase enzymes in in vivo assays [228]. Furthermore, propolis extract reduceed blood glucose levels in a dose-dependent manner [229–237]. The inhibitory impact of numerous triterpenes isolated from propolis such as cycloartenol, ambonic acid, mangiferonic acid and ambolic acid on α-glucosidase was investigated by Pujirahayu et al. [238]. Mangiferonic acid showed the highest inhibitory impact on α-glucosidase with an IC_50_ of 3.46 µM [238]. Moreover, propolis and bee pollen extracts ameliorated the insulin resistance [233] as well as the α-glucosidase and α-amylase [239] activity, helping to reduce blood glucose.

The insulin receptor, acting like a tyrosine kinase receptor, shows a wide range of function in human body. Insulin receptor signal pathway helps to regulate glucose transporter in membranes of several cells including hepatocytes, adipocytes, and skeletal muscle cells for energy metabolism [240]. Insulin resistance can be caused by disruptions in various signaling pathways. Modulating insulin receptor signaling at various points along the intracellular route can improve insulin responsiveness in a variety of tissues and, reduce insulin resistance [240]. Several studies demonstrated that natural compounds may modulate the insulin receptor signal with promising results. Liu et al. [241] revealed that propolis with its main compounds galangin and pinocembrin can modulate insulin receptor signaling through regulating Akt/mammalian target of rapamycin (mTOR) signaling pathway [241]. Furthermore, these two phytochemicals attenuated insulin resistance via increasing the insulin sensitivity, Akt, and glycan synthse kinase-3 (GSK3) β and decreasing insulin receptor substrate 1 (IRS-1) phosphorylation. Propolis inhibited the expression of glucose 6-phosphatase (G6Pase) by inhibiting the autophosphorylation of of GSK3α and β, which are involved in the activation of GSK3 [242]. In diabetes, IRS-1 phosphorylation has been established to be related to insulin signal transduction, hence galangin, and pinocembrin reinstate the sensitivity of insulin receptors and diminish insulin resistance [241]. Another study reported that CAPE increased the IRS-1. On the contrary, p-JNK, p-NF-B p65, and nuclear translocation of p-NF-B p6 were suppressed [243]. Tectochrysin (5-hydroxy-7-methoxyflavone) is a bioactive substance from propolis, which exerted hypoglycemic effect by blocking the α-glucosidase enzyme [244]. Besides, propolis with chitosan polyacrylic (CS-PAA) nanoparticles diminished blood glucose levels [245]. Finally, propolis reduced body cholesterol and triglycerides levels and increased peroxisome proliferator activated receptor alpha (PPAR-α) protein expression in the liver [246]. In mice, an ethanol extract of propolis showed anti-hyperlipidemic and antioxidant activity and exhibited protective action on the cardiovascular system and hepatorenal functions [247]. Anvarifard et al. [248] recently reviewed the cellular mechanisms of Propolis on preserving renal functions. Antidiabetic effects of propolis extracts and their isolated bioactive compounds are shown in Table 9.

**Table 9:** Antidiabetic effects of propolis extracts (PE) and their isolated bioactive compounds

Extract/Compound	Concentration	Study level	M/A	References
PE	IC_50_ 3.77 µM	In vitro	α-Glucosidase inhibition	[220]
Chihuahua propolis	300 mg/kg/day, orally, 15 days	In vivo	Suppress Blood glucose levels	[234]
Astrapterocarpan, medicarpin, 8-prenylnaringenin	8.0, 5.0 and 4.0 mg/g	In vivo	α-Amylase and α-glucosidase inhibition	[228]
Propolis and bee pollen extracts	100–200 mg/Kg b.w., orally, 16 weeks	In vivo	Reduction of blood glucoseand insulin resistance	[233]
PE	300 mg/Kg b.w., orally, 4 weeks	In vivo	Reduction of fasting blood glucose	[232]
PE	50–100 mg/Kg b.w., orally, 15 days	In vivo	Reduction of blood glucose	[231]
PE	300 mg/Kg b.w., orally, 15 days	In vivo	Reduction of blood glucose	[234]
PE	IC_50_ 70.79 µg/mL	In vitro	α-Glucosidase inhibition	[227]
PE	IC_50_ 0.09 mg/mL	In vitro	α-Amylase and α-glucosidase inhibition	[239]
PE	200–300 mg/Kg b.w./day, orally, 28 days	In vivo	Reduction of blood glucose, conservation of normal pancreatic cell architecture	[235]
PE	100 mg/Kg b.w., oral intubation, twice daily, 8 weeks	In vivo	Reduction of fasting blood glucose, reduction of glycated hemoglobin; restoration of hepatorenal functions	[236]
PE	200 mg/Kg b.w., orally, 5 weeks	In vivo	Reduction of serum glucose, reduction of oxidative stress parameters	[237]
PE	100–300 mg/Kg b.w., orally, 8 weeks	In vivo	Reduction of plasma level of insulin and HOMA-R index of insulin resistance	[229]
PE	1 mL/100 g, intragastrically, twice daily, 8 weeks	In vivo	Reduction of blood glucose, reduction of fructosamine, malonaldehyde and nitric oxide	[230]
PE	IC_50_ 4.7 mg/mL	In vitro	Maltase and α -amylase inhibition	[400]
PE	1–2% *w*/*w*	In vivo	Reduction of cholesterol, triacylgycerol and ALT	[449]
PE	70 µL of 85% PE/animal, 60 days	In vivo	Increase of plasmatic HDL, prevention of LVH and arterial atherogenesis	[450]
PE	160 mg/Kg b.w., 14 weeks	In vivo	Reduction of total cholesterol and triglycerides, without any effect on HDL, decrease of atherosclerotic lesion development in aortic root	[451]
PE	250 mg/Kg b.w./day, orally, 4 weeks	In vivo	Normalisation of lipid profile/down-regulation of VCAM, FGF, VEGF and MMP-9 gene expression	[247]
PE	75 mg/Kg b.w., orally	In vivo	Reduction of total cholesterol, LDL and triglycerides	[266]
PE	0.05–0.5% *w*/*w*, orally, 8 weeks		Reduction of cholesterol and triglycerides/increase of PPARα protein level in the liver	[246]
CAPE	60 ng/mL	In vitro and in vivo	Increase of p-Akt and p-insulin receptor substrate (IRS)-1, inhibition of p-JNK, p-NF-κB p65, and nuclear translocation of p-NF-κB p6	[243]
PE conjugated with chitosan polyacrylic (CS-PAA) nanoparticles		In vivo	Suppress blood glucose levels	[245]
PE	300, 600 mg/Kg b.w., orally, 4 weeks	In vivo	Reduce glucagon, FBG level, and improve insulin and islet of Langerhans regeneration	[452]
Tectochrysin	60 μl/mL	In vitro	α-Glucosidase inhibition	[244]
PE	200, 600 mg/Kg b.w./day, orally, 6 weeks	In vivo	Decrease of glucose level	[453]
PE	100 g/Kg b.w., oral intubation, twice daily, 8 weeks	In vivo	Decrease of glucose level	[236]
PE	240 μM	In vitro	Decrease the of G6Pase expression by inhibiting the autophosphorylation of GSK3α and β, which are involved in the activation of GSK3	[242]

### Wound healing activity

Wound healing is a complex, multi-step process that can be hampered by a variety of internal and environmental causes [249]. Skin is one of the most prominent part of human body and acts as first line defence system. It can be pertubated by several factors including inflammation and bacterial infections and, these events often require long therapy to achieve a perfect healing [250]. According to recent findings, propolis aids wound healing in a time-dependent manner. The immunomodulatory [10], antimicrobial [251], antioxidant [252], analgesic, and anti-inflammatory [253] properties of propolis might account for accelerating the wound healing [254]. Vascular endothelial growth factor (VEGF), is a growth factor related to endothelial cell growth and migratory activation. VEGF promotes the growth of fibroblasts [255, 256], which play a pivotal role in the wound healing process [257]. Collagen deposition is an integral part of the healing process. It has been proved that propolis contributes also to increase the cell influx and collagen deposition [258].

Propolis has been shown in recent research to have a significant wound healing impact by up regulating the healing process at the tissue level. The preventive impact of propolis is mainly attributed to aminoacids, flavonoids, phenolic acids, terpenes, and vitamins [259]. Propolis extract displayed protective activity by regulating antioxidant-related genes including heme oxygenase 1 (HO-1), GCLM, GCLC in wounded tissue [260]. Propolis bioflavonoids improved immunity and induced the production of interferon by white blood cells or lymphocytes. In a rat experiment model, propolis also activated the thymus, the immune system's master gland, and healed wounds. Furthermore, it has a greater wound healing activity than silver sulfadiazine when used as a skin cream [261].

Mast cells, characterized by inflammatory, proliferative, and remodeling phases, play important roles in wound healing. Mast cell enhance skin wound healing throughout the release of vasochemical madiators such as histamine, proteases, TNF-α and arachidonic acid metabolites [262], and promote the anticoagulant activity. Furthermore, mast cell attach leukocyte to the injured areas, and with macrophages, contribute to phagocytosis and debridement [262]. Mast cells also help to enhances angiogenesis, fibroplasia and re-epithelialization [263]. Propolis used topically reduce the number of mast cells, favouring wound healing [264]. Isolated bioactive constituents from propolis, like CAPE and other active compounds reduced the levels of mast cell and improved surgecial wound during acute inflammation phase [263]. Furthermore, CAPE attenuated histamine release and inflammatory cytokines in wounded tissue [265, 266]. Propolis and its components, such as chysin and kaempferol, have anti-inflammatory properties [267]. Chrysin have the ability to inhibits inflammatory cytokines gene expression such TNF-α, IL-1, IL-4, and IL-6 in mast cells through NF-kB and caspase-1 related mechanistic pathways [267, 268]. Moreover, kaempferol can also inhibit degranulation and cytokine production by the activated mast cells [267] and improved wound healing capacity of propolis in experimental wound healing model.

Because oxidative stress occurs after wound, the antioxidant activities of propolis could favour tissue repair [269, 270]. Some studies reported that release of pro-oxidant mediators, such as reactive oxygen and nitrogen species, is linked to severe burns [270, 271]. Free radicals in burns disrupt the functionality of cellular membranes and intracellular oganelles, and numerous inflammatory signaling processes, reducing the wound healing activity [271]. Hence, antioxidant compounds represent promising therapeutic agents for attenuating burn related injuries [270]. In several studies, it has been demonstrated that propolis exert antioxidant qualities, making it a viable alternative for regulating burn wound healing [71, 74, 272, 273]. Okinczyc et al. [273] reported that propolis from Eurasian areas have potentiall antioxidant activity. Propolis exert antioxidant activity in burn wound healing process due to its flavonoid and phenols content [274]. Mohammadzadeh et al. [274] stated that there is a strong relationship between the chemical composition of propolis and its antioxidant activity. The flavonoids (including flavones, flavonols, flavanones and dihydroflavonols) and other phenolics (mainly cinnamic acids and their esters) are able to scavenge free radicals [247, 275]. Polyphenolic compounds of propolis displayed a protecting role by reducing the proteins break down, and maintaining the integrity of cellular membrane decreasing peroxidation and hemolysis [272]. CAPE with some other polyphenols can upregulate the expression of aminopeptidase (AMP) gene, which helps re-epithelialize skin cell in chronic wound [276, 277] and reduce lipid peroxidation reducing the DNA and protein breakage [71]. In addition, propolis extract rich in galangin, has the ability to inhibit superoxide anion production and suppress oxidative stress in wound injuries [71].

In association with nanoparticles (polymeric nanofibrous wound dressing of polyvinyl alcohol (PVA), propolis, showed significant potential tissue regeneration [278]. Furthermore, biogenic silver nanoparticles (Bio AgNP) with propolis showed antibacterial activity against Gram– and Gram + bacteria stimulating fibroblast cell proliferation and heal wound in surgical sutures [279]. In a study, propolis extract incorporated in a biocompatible polyurethane-hyaluronic acid (PU-HA) nanofiber in wound dressing, displayed a potent activity favouring the fibroblast cells adhesion, deposition of collagen fibers and cell proliferation in wounded areas [280, 281]. A propolis mixture of two different geographical location evidenced better potential effect than individually on wound healing through re-epithelization process in rat chronic wound by bacterial inhibition [282]. It has been demonstrated that topical application of propolis paste (30%) showed a beneficial effect on cutaneous wound healing in dog experimental model [283]. Furthermore, propolis has dermal construction effect evidencing significant reduction in the wound surface area [30].

CAPE, a component of propolis, has immunosuppressive properties in T-cells, which play a significant role in the development of inflammatory disorders. CAPE also reduced both the expression of IL-2 gene and the production of IL-2 in activated T-cells [283]. Propolis also includes antiviral compounds such as 3-methyl-but-2-enyl caffeate, isopentyl ferulate, and moronic acid [5]. Additionally, together with ascorbic acid, propolis promoted wound healing in diabetic patients through dermal regeneration in a rat model [284].

Re-epithelialization is a critical phase in wound healing involving keratinocyte migration and proliferation [285]. Propolis along with its chemical constituents increases aquaporin-3 (AQP3) gene expression [254]. AQP3-facilitated water transport plays a pivotal role in cell migration and hyperproliferation and accelerates the healing of cutaneous wounds stimulating epidermal keratinocytes [254]. Therefore, in conclusion propolis from different countries heal wounds by regulating a plenty of signalling pathways including mast cell regulating via and AQP-3, VEGF, NF-kB and caspase-1 pathways.

### Antiviral activity

The world has witnessed the outbreak of several detrimental viral epidemics and/or pandemics since the creation of human civilization that have established a remarkable spotlight in the sectors of public health, education, and economy. The substantive example is the ongoing Covid-19 pandemic that nobly alters human lifestyle and creates a frenzied challenge for researchers to deliver safe, tolerable, and effective treatment strategies, including natural product therapy [286], experimental drug candidates [287], and vaccines [287]. Various researchers in most of the countries are continuously trying to overcome this hectic challenge by computational technologies to determine predictable therapeutic responses at fast along with cost and time saving matters [288–290]. Regarding all kinds of viral issues, the alternative and complementary ethnomedical practices (i.e., plants, natural products, and natural compounds) in various holistic approaches led to the greatest concern in the direction of the therapeutic development throughout the world [286]. At the same time, to establish an appropriate therapeutic candidate, some non-clinical and pre-clinical studies before the clinical studies must be established and find out the satisfactory results that can lead to the clinical trial [291]. Based on these perspectives, some researchers began to investigate the antiviral properties of propolis and several isolated compounds.

Various propolis extracts (e.g., aqueous, ethanolic, and hydroalcholic) at controllable laboratory condition have been found to exeret substantial antiviral activity against several types of viruses such as *Herpes simplex* virus type 1 and 2 (HSV-1 and HSV-2), *Canine distemper* virus, *Human rhinovirus* type 2, 3 and 4 (HRV-2, HRV-3 and HRV-4), *Influenza virus* type A and B, *Parainfluenza* virus, *Human immunodeficiency virus* (HIV), and *Adenovirus* [292, 293].Research findings demonstrated that aqueous and ethanol propolis extracts exhibited a strong antiviral activity against HSV-1 [294] and HSV-2 [295] on RC-37 cells by affecting viral plaque formation and viral infection cycle. On the other hand, 0.5% aqueous extract of propolis showed potent antiviral activity against HSV-1 infected rats and rabbits thorough prevention of virus absorption into the host and inhibition of an internal step(s) during the viral replication cycle [296]. In addition, ethanol Mexican propolis extract exerted antiviral activity against *Canine distemper* virus through the inhibition of the relative expression of the virus nucleoprotein gene [297]. It has been cited that a 5% alcoholic solution of propolis strongly prevented influenza viral proliferation in experimental influenza virus-infected mice [298]. Furthermore, the Brazilian propolis ethanolic extract adequately blocked the entry of viruses (HRV-2, HRV-3, and HRV-4) into the experimental HeLa cells protecting them from the virus destruction and reducing virus replication [299].

Some flavones and flavonols (especially galangin, kaempferol, and quercetin) extracted from propolis have been cited to exert antiviral activity against HSV-1 in variable doses [300]. Another investigation by Amoros et al. [301] reported that 3-methyl-but-2-enyl caffeate showed anti-HSV-1 activity through preventing the viral DNA synthesis. Finally, recent studies, showed that both propolis and its constituents had potential efficacy against SARS-CoV-2 by modulating multiple pathogenic pathways [302–311]. Molecular docking studies have demonstrated high binding affinities of propolis compounds to several SARS-CoV-2 proteins, including 3C-like protease, papain-like protease, RNA-dependent RNA polymerase, the receptor-binding domain of the spike protein and helicase. Moreover, these studies showed a high affinity against the viral target angiotensin-converting enzyme 2 (ACE2). Among these compounds, the most promising one was the retusapurpurin A, which showed the best affinity for the most viral targets mentioned above inhibiting the viral entry by forming hydrogen bonds with aminoacid residues within viral and human target proteins [312]. Table 10 shown the antiviral activity of propolis water, ethanol and hydroalcholic extracts and isolated compounds.

**Table 10:** Antiviral activity of propolis water, ethanol and hydroalcholic extracts (WE, EE and HAE, respectively) and isolated compounds

Extract/Compound	Concentration /Dose	Test system	Results/Mechanisms	References
WE and EE	0–120 pfu (%)	HSV-1 on RC-37 cells	Affects viral infection cycle	[294]
WE and EE	0.00005–0.005 pfu (%)	HSV-2 on cells RC-37	Inhibition of HSV-2 plaque formation (IC_50_ 0.0005% and 0.0004%, respectively)	[295]
0.5% WE	*_*	Rat and rabbit infected with HSV-1	50% inhibition of HSV infection	[296]
Mexican EE	250 µg/mL	*Canine distemper virus*	Inhibition of the virus nucleoprotein gene expression	[297]
5% HAE	_	*Influenza virus* in mice	Prevents influenza viral proliferation	[298]
Brazilian EE	50–100 µg/mL	HRV-2, HRV-3, and HRV-4 in HeLa cells	Block virus entrance into the cells, avoiding virus destruction and replication	[299]
Galangin, kaempferol, quercetin	0.05–1.0 µM	HSV-1	Reduce the viral titer by 2log_10_	[300]
3-methyl-but-2- enyl caffeate	6.25, 12.5, 25 and 50 µg/mL	HSV-1	Reduce the viral titer by 3log_10_ and viral DNA synthesis by 32-fold	[301]
CAPE	5 µM	HTLV-1	Inhibits NF-kB activation during in vitro infection	[454]
Green propolis EE	5 mg	Suid herpesvirus type 1 (SuHV-1)	Increases humoral and cellular response in mice immunized with SuHV-1	[455]
Quercetin	5–25 µM 50 µM 1–50 µM	Rhinovirus (RV) Hepatitis C virus (HCV) Hepatitis B virus (HBV)	Inhibits rhinovirus replication in vitro and in vivo; Prevents up-regulation of diacylglycerol acyltransferase (DGAT) required for HCV replication in vitro; Decreases heat shock proteins and HBV transcription levels in vitro	[456] [457] [458]
Caffeic acid	IC_50_ = 3.9 µM—100 mg/kg b.w. *per os*; 4 mM IC_50_ = 7.2 and 8.5 µM on NA1 and NA2, respectively	HBV Influenza A virus (IAV) IAV	Inhibits HBV-DNA replication in vitro and in vivo*;* Inhibits replication IAV in vitro; Inhibits IAV activity through neuraminidases (NA) in vitro	[277] [459] [460]
Rutin	Binding energy -8.97 kcal/mol (in silico)	SARS-CoV-2	Inhibitory potential on ACE2	[302]
Anatolia propolis EE, hesperetin (HE), pinocembrin (PI), CAPE	IC_50_ = 0.57–1.14 µL, 16.88 mM, 29.53 mM, 79.09 mM; Binding energy to spike S1 protein: -7.28, -7.54, 7.17 respectively for HE, PI and CAPE	SARS-CoV-2	Binds spike S1 protein and ACE-2 receptor as both in vitro and in silico studies	[303]
Optimized liposomal formula of propolis, rutin and CAPE	IC_50_ = 1.18 mM, ICM score: -92.8 and -67.8 agaist 3CL protease; ICM score: -94.3 and -77.8 agaist S1 spike protein	SARS-CoV-2	Bind COVID-3CL protease and S1 spike protein; inhibit the viral replication	[304]
Glyasperin A and broussoflavonol F	Binding affinity -7.8 kcal/mol	SARS-CoV-2	Bind SARS-CoV-2 main protease	[305]
Withaferin-A, Withanone and CAPE	Binding affinity: -5.6, -4.3 and -6.20 kcal/mol, respectively	SARS-CoV-2	Bind transmembrane protease serine 2 (TMPRSS2) in molecular docking studies	[306]
Withanone and CAPE	Binding affinity: -4.42 and -4.79 kcal/mol, respectively	SARS-CoV-2	Bind SARS-CoV-2 protease M^pro^	[307]
3'-Methoxydaidzin (MD), neoblavaisoflavone, methylophiopogonone A and genistin	Binding affinity: -7.7 for MD and -7.6 kcal/mol for other compounds against M^pro^; -8.1, -8.2, -8.3, and -8.3 kcal/mol, respectively against spike protein S2	SARS-CoV-2	Bind main protease (Mpro) and spike protein subunit 2 (S2)	[308]
Glyasperin A, broussoflavonol F, sulabiroins A, (2S)-5,7-dihydroxy-4'-methoxy-8-prenylflavanone and isorhamnetin	Docking score of -10.8, -9.9, -9.5, -9.3 and -9.2 kcal/mol respectively	SARS-CoV-2	ACE-2 inhibitors	[309]
2,2-Dimethyl-8-prenylchromene, Artepillin C, 3-Prenyl cinnamic acid allyl ester, Isocupressic acid, 13C-symphyoreticulic acid, ellagic acid, syringic acid, caffeic acid phenethyl ester, p-coumaric acid, hesperetin, naringenin, kaempferol, quercetin, chrysin	Binding scores: -5.6—-7.8 kcal/mol against M^pro^; -5.3—-6.4 kcal/mol against RdRp	SARS-CoV-2	Main protease (M^pro^) and RNA-dependent RNA polymerase (RdRp) enzymes	[310]

### Antidepressant and anxiolytic-like effects

Diverse estudies have been reported the effects of propolis in the central nervious system (CNS) as a neuroprotector and in amelioraitng alterations in behavior. Propolis extract (10–50 mg/kg) improved the locomotor activity and exert anxiolytic-and antidepressant effects in vivo like diazepam, as observed by in vivo open field (OF), elevated plus-maze (EPM) and forced swimming tests, respectively [313]. Moreover, propolis extract decreased the NO release in response to swim stress induced in experimental model [313]. Theses pharmacological effects are mainly attributed to its polyphenolic constituents such as flavonoids, phenolic acids and terpenes [314–316]. In the OF test, Lee et al. [317] revealed that propolis extract had an antidepressant-like effect without affecting locomotor function or spontaneous movement in a dose-dependent manner. Furthermore, they hypothesized that the antidepressant effect may be mediated by an elevation in hippocampal glucocorticoid receptor (GR) activity, which modulates the hypothalamic–pituitary–adrenal (HPA) axis by attenuating corticosterone response and, reducing c-fos neurons in brain tissue and lipid peroxidation in CNS. Moreover, CAPE, an active biocomponent of propolis, down-regulated c-fos levels in neurons, which relate with antidepressant-like effect [318]. Additionally, CAPE knocked down the expression of p38/MAPK signaling pathway, improving GR function and exerting antidepressant effect [69]. Chrysin, another active chemical constituents of propolis, exerted an antidepressant activity in vivo by elevating the brain-derived neurotropic factor (BDNF) and nerve growth factor (NGF), Na^+^, K^+^-ATPase, non-protein thiol (NPSH), corticosterone level [319] and down-regulated ROS levels [320]. Moreover, chrysin, as already demonstrated for the CAPE, exhibited antidepressant modulation via regulating the HPA axis [319]. Finally, chrysin contributed to reduce the stress by decreasing the release of several pro-inflammatory cytokines such as TNF-α, IL-1β and IL-6 [319].

A recent study had revealed that propolis extract drastically reduce cortisol (CORT), adrenocorticotropic hormone (ACTH) and MDA and elevate superoxide dismutase (SOD) levels in a stressed mice model. Therefore, this study clearly demonstrated that propolis extract exerted anti-anxiety properties through the inhibition of the hyperfunction of the HPA axis and stimulation of antioxidation processes in nervous system [321]. In the OP test, however, anxiolytic-like agents led to an increase in central locomotion [322], and a diminution in grooming [323]. Propolis, on the contrary, increased locomotion in OF test [317, 321], thus the anxiolytic effect of propolis remains a bit controversial and further studies are needed to corroborate this hypotesis.

### Apitherapy

**Fig. 3:**
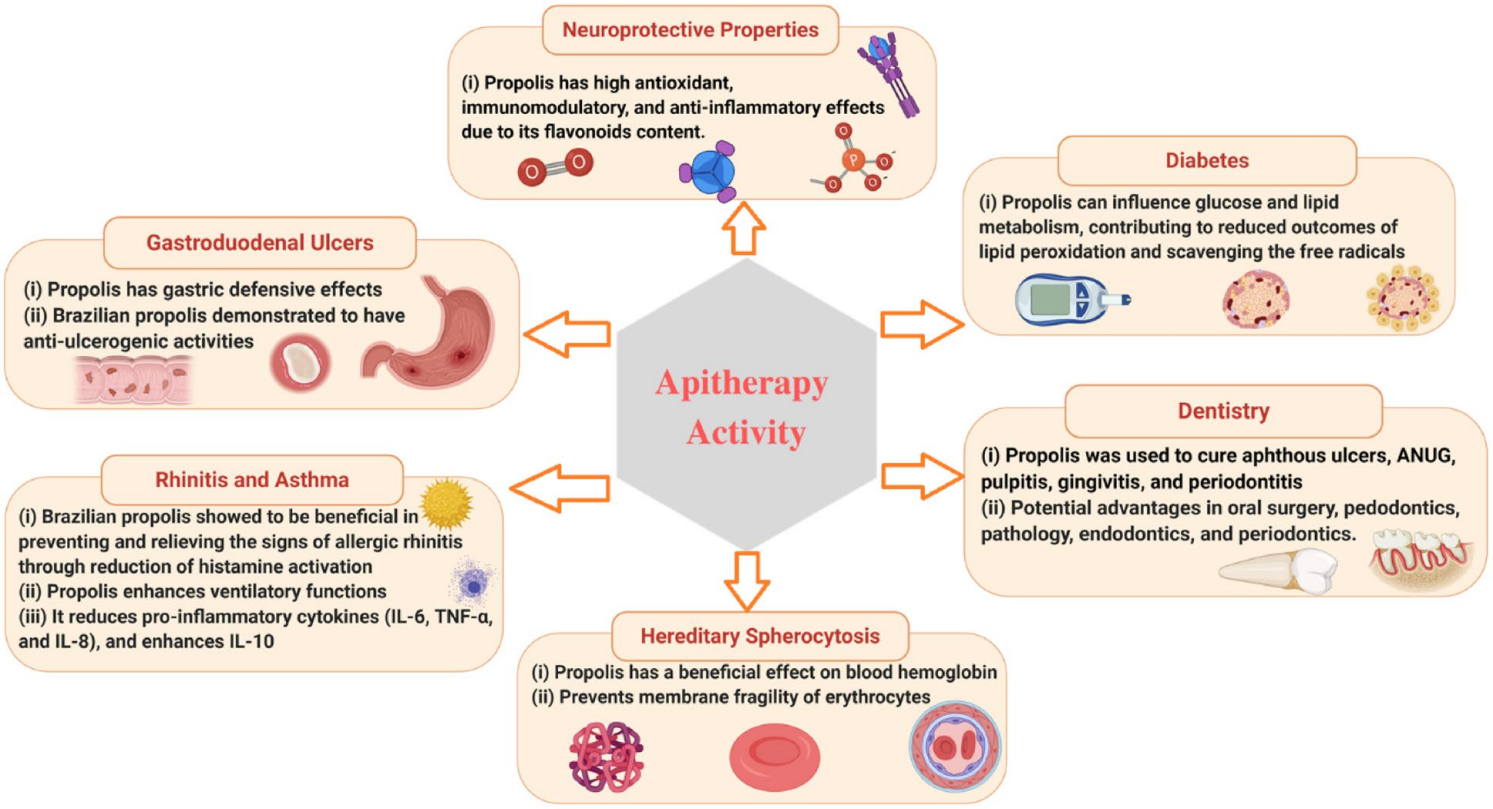
Apitherapy: therapeutic effects of propolis

Apitherapy is a set of treatments aimed at recovering well-being, both in the human and veterinary fields, with products collected, processed, and secreted by bees including honey, propolis, pollen, bee venom, and royal jelly. Bee products are conventional drugs in several countries. The apitherapy has its origin in the pharmacy of Antique Egypt about 6000 years ago. Also, the ancient Greeks and Romans have used the ingredients of bees for various types of remedies [324, 325] (Fig. 3).

Propolis possess several helath properties including antiallergic, antimicrobial, antioxidant, cardioprotective effects, anti-cancer, anti-inflammatory, antiulcer, antitumor, hepatoprotective, neuroprotective and antidiabetic effects. The efficacy of neuroprotective flavonoids primarily depends on their high antioxidant, immunomodulatory and anti-inflammatory functions [217, 326–330]. In addition, an in vitro study showed that green propolis enhanced the differentiation, mitigation, and proliferation of stromal cells in bone marrow [331]. The therapeutic properties of the bioactive substances in propolis have been investigated in recent drug screening studies [290, 332, 333]. In dentistry, propolis was used to cure aphthous ulcers, acute necrotizing ulcerative gingivitis, pulpitis, gingivitis, and periodontitis. Because of its medicinal and biological effects, propolis investigation has been expanded. Present dental propolis study includes a wide variety of areas and emphasizes the anti-inflammatory as well as antimicrobial activities of this bee product, notably in oral surgery, pedodontics, pathology, endodontics, and periodontics [334].

Diabetes is a metabolic condition defined by hyperglycemia as well as glycosuria attributed to complete or relative insulin insufficiency. Oxidative stress has been involved in the improvement of diabetic nephropathy. Most investigators showed the streptozotocin (STZ) exposure as a diabetes-inducing factor. One study revealed that propolis extract induced pancreatic β-cells survival against STZ toxicity in rats [335]. Also, researchers found that the administration of water or ethanolic extract of propolis for seven weeks to STZ-induced diabetic rats influenced glucose and lipid metabolism, contributing to reduced outcomes of lipid peroxidation and scavenging the free radicals in diabetic rats [230]. However, it has been documented that the treatment with propolis for 8 weeks to 15% fructose-exposed rats, led to a decrease in body weight as well as insulin concentration without influencing blood glucose concentration [229].

Gastroduodenal ulcers may arise from the unbalance between inflammatory and protective processes in the stomach, such as alteration of the mucosal barrier, acid–pepsin secretion, cellular regeneration, mucus secretion, and epidermal growth factors [336]. It has been reported that propolis has gastric protective effects in mice at several doses (e.g., 50, 250, or 500 mg/kg) [337]. Also, the Brazilian propolis (50 mg/kg or 250 mg/kg) showed anti-ulcerogenic activities in different models [338]. Moreover, the authors investigated the impact of the essential oil derived from the most significant botanical source (*Baccharisdracun culifolia*) of the Brazilian green propolis on gastric ulcers, showing that it may be a successful therapeutic agent for the development of novel phytotherapeutic approach to treat gastric ulcers [339]. Recently, Boeing et al. [340] observed that the hydroalcoholic extract of red propolis from Northeastern Brazil displays gastroprotective property against ethanol/HCl-induced damage in mice.

Rhinitis is a form of sinusitis characterized by nasal congestion, purulence and sneezing due to bacterial or viral infections. It is a world health issue that affects people's work, education, sleep, and social lives [341, 342]. Brazilian propolis showed to be beneficial in preventing and relieving the signs of allergic rhinitis through the reduction of histamine release. The study showed that a single administration of 1 g/kg propolis was ineffective in preventing antigen-induced nasal rubbing and sneezing, but substantial improvement was described after continuous administration for 2 weeks at this dose [341]. Another respiratory tract disease to consider is asthma as an inflammatory disease of the airways related to overexposure to allergens characterized by congestion, inflammation, as well as repetitive movements and remodeling [343]. Khayyal et al. [344] observed that the daily administration of an aqueous sample of propolis (13%) to patients with mild to severe asthma for two months led to a decrease in night-time attacks and enhanced ventilatory functions. These improvements seemed to be related to a decrease in the release of pro-inflammatory cytokines (IL-6, TNF-α, and IL-8) and an increase of the anti-inflammatory cytokine IL-10, [344].

Hereditary spherocytosis (HS) is an aberrant disease of red blood cells (RBCs). The disease is triggered by a mutation in the genes responsible for the production and maintenance of hemoglobin. Ordinary red blood cells are biconcave disks, and those with HS have a spherical shape (spherocytosis) [345–347]. A recent study evaluated the influence of the propolis extracts on the ability of the red blood cell membrane to retain ions. It has been observed that propolis had a beneficial effect on blood hemoglobin. This result has been attributed to the high content of phenolics in the propolis. The findings of the in vitro evaluation suggested that the membrane fragility improves in a patient's RBCs, and tat the protective advantage of propolis was attributable to its antioxidant properties [348].

## Propolis as food preservative

The utilization of propolis in the human diet should be increased to take advantatge of the benefits of its biological actions on human health. It also meets consumer demand for natural antioxidants and antimicrobials rather than synthetic food additives. Therefore, adding propolis to food products as a natural food additive and increasing food quality has become a trending topic [332]. Many of the compounds found in propolis have been utilized as functional food additives and are considered to be safe for human health [13, 30]. However, although propolis is generally considered safe, some people may experience side effects such as dermatitis [11].

Propolis is commonly used in food formulations such as oils, dairy, meat, seafood, fruits, and juice to extend the shelflife, to reduce lipid oxidation, and to provide health advantages to consumers [349]. Vargas-Sánchez et al. [350] observed that propolis extracts can be applied to increase lipid oxidation stability and to prevent microbial growth on beef patties during cold storage. Cottica et al. [351] reported that adding propolis aqueous extract to dairy beverages resulted in increased antioxidant capacity and lower aldehyde generation during storage with exposure to light. Considering that the use of propolis to coat quail eggs protected egg quality metrics, its use may help delay the loss of quality during storage [352].

## Conclusions and future directions

Propolis is a unique natural remedy, used since ancient times in traditional medicine, whose pharmacological properties are in constantly updated. The present study summarized the known chemical pofile of propolis until to date collecting data from multiple databases and shelding light also to the phytochemcal differences due to the origin and grow pedo-climatic conditions. Summarizing, about 500 chemical compounds have been described till date, mainly belonging to the flavonoids, terpenoids and phenolic acids classes. However, other secondary metabolites like alkaloids and iridoids were overlooked but have not been yet isolated. Available scientific literature confirms the efficacy of propolis and its bioactive components against bacterial and viral infections and cancer. However, the big problem about the study of therapeutic applications of propolis is its varied composition that depends on the flora of the area, the time of collection, weather conditions, environmental pollution, inclusion of polluting waxes, among others. This makes it difficult to define propolis for medicinal use since its composition varies greatly. Added to the above is the use of different extraction methodologies and solvents used in the different preclinical studies. To solve these problems in the future, standardization should be worked on by establishing exact chemical tests in order to control quality. Currently there is a problem of quality control in propolis products on the market. Apart from its innumerable health uses, some of which have been described recently, such as the possibility of using propolis extracts and its bioactive compounds against SARS-COV-2 infection, propolis can also be used for food preservation. However, further studies are needed to substantiate these claims, in particular, clinical studies with standardized extracts, in order to guarantee the reproducibility of the health properties observed and to translate on humans what observed in the numerous in vitro and animal studies.

## Data Availability

Yes.
